# Disrupted calcium dynamics and electrophysiological activity in the stratum pyramidale and hippocampal alveus during fear conditioning in the 5xFAD model of Alzheimer’s disease

**DOI:** 10.3389/fnagi.2025.1550673

**Published:** 2025-08-20

**Authors:** Alexander Erofeev, Egor Vinokurov, Anastasia Bol’shakova, Ilya Bezprozvanny, Olga Vlasova

**Affiliations:** ^1^Laboratory of Molecular Neurodegeneration, Graduate School of Biomedical Systems and Technologies, Institute of Biomedical Systems and Biotechnology, Peter the Great St. Petersburg Polytechnic University, Saint Petersburg, Russia; ^2^Laboratory of Molecular Neurobiology, Pavlov Institute of Physiology, Russian Academy of Sciences, Saint Petersburg, Russia

**Keywords:** Alzheimer’s disease, 5xFAD, calcium imaging, miniscope, LFP, hippocampus, hippocampal alveus, fear conditioning test

## Abstract

Alzheimer’s disease (AD) is a neurodegenerative disorder that leads to progressive cognitive decline and significant disruptions in hippocampal neural networks, critically impacting memory and learning. Understanding the neural mechanisms underlying these impairments is essential for developing effective therapies. The 5xFAD mouse model, known for progressive neurodegeneration and cognitive deficits, provides a valuable platform for investigating associative learning and memory impairments related to AD. However, the *in vivo* electrophysiological state of the hippocampal alveus in 5xFAD mice during learning and memory formation remains poorly understood. Here, we performed *in vivo* one-photon calcium imaging of CA1 hippocampal neurons with wireless electrophysiological recordings from the hippocampal alveus in freely moving 5xFAD mice to explore specific neural alterations during a fear conditioning test. Our results demonstrate significant deficits in the learning and memory capacities of 5xFAD mice, showing impairments in hippocampal-dependent contextual and tone-associated memory retrieval, along with disrupted calcium dynamics and impaired electrophysiological activity in the hippocampal alveus. These findings reveal patterns of network dysregulation associated with AD. These findings enhance our understanding of the specific neural dysfunctions contributing to the cognitive decline associated with memory loss in AD and emphasize the value of applying *in vivo* methods to elucidate neurodegenerative mechanisms. This approach provides a foundation for future studies on AD pathophysiology and may inform the development of targeted therapeutic strategies to mitigate memory impairments in AD.

## 1 Introduction

Alzheimer’s disease (AD) is a neurodegenerative disorder that is characterized by the progressive loss of memory and other cognitive functions. This ultimately results in an inability to perform the activities of daily living and routine tasks. It is the most prevalent form of dementia, currently affecting over 50 million individuals globally (Gustavsson et al., 2023), with this number expected to rise as the population ages, thereby increasing the burden on healthcare systems and society (Brookmeyer et al., 2007). The pathological hallmarks of Alzheimer’s disease (AD) include extensive neuronal death and synaptic dysfunction, particularly in the hippocampus, a region that is crucial for learning and memory (Rao et al., 2022). The CA1 area of the hippocampus is of particular importance for cognitive functions such as spatial navigation (O’Keefe and Dostrovsky, 1971; O’Keefe and Nadel, 1979; Sun et al., 2019). Neurodegeneration in AD also affects other parts of the brain, such as the hippocampal alveus, which is a thin layer of white matter made up of the axons of the pyramidal cells in the hippocampus (Boutet et al., 2014; Parmar et al., 2018).

Mouse models of Alzheimer’s disease are of great value to the scientific community, facilitating the analysis of disease pathogenesis, the evaluation of potential therapeutic agents, and the investigation of the molecular mechanisms underlying the disease (Webster et al., 2014). Among these models, the 5xFAD transgenic mouse line plays a pivotal role in Alzheimer’s disease research, offering insights into disease pathogenesis and enabling the evaluation of therapeutic interventions (Webster et al., 2014). This model overexpresses mutations associated with familial Alzheimer’s disease, resulting in early onset and rapid progression of the disease, and mimicking severe pathological features such as amyloid plaque formation and synaptic degeneration (Oakley et al., 2006; Jawhar et al., 2012; Forner et al., 2021; Liu et al., 2023).

A comprehensive study of Alzheimer’s disease pathology requires the utilization of a range of experimental methodologies, as exemplified by the 5xFAD mouse model. These encompass genetic, histological and biochemical techniques (Oblak et al., 2021; Prajapat et al., 2023). Additionally, behavioral tests, *in vivo* calcium imaging and electrophysiological studies in freely moving animals provide valuable data, simulating conditions that closely resemble natural environments. This approach facilitates a more profound understanding of the neurodegenerative mechanisms involved in Alzheimer’s disease (Morrone et al., 2022).

The fear-conditioning test is frequently used in research animals, particularly rodents such as mice and rats, to assess associative learning and memory. This behavioral technique evaluates the ability of animals to learn and remember the association between a neutral stimulus (e.g., sound or light) and an aversive stimulus (typically an electric shock) (LeDoux, 2000). Following subsequent exposure to the context or cue without the aversive stimulus, mice will exhibit freezing behavior, indicative of a conditioned fear response (Shoji et al., 2014). The fear-conditioning test is renowned for its simplicity, high reproducibility, and effectiveness. It is also one of the few methods compatible with *in vivo* calcium imaging and electrophysiology. When combining the fear-conditioning test with electrophysiological recordings, there is a risk of artifacts arising from the application of electrical shocks. To minimize these artifacts, temporal separation is applied between shock delivery and recording intervals, along with high-pass filtering to ensure accurate data with minimal distortion (Islam et al., 2021). A number of studies employing the fear-conditioning test have confirmed the onset of cognitive impairments in 5xFAD mice beginning at the age of 5 months (Kimura and Ohno, 2009; Kaczorowski et al., 2011; Bouter et al., 2014; Dinkins et al., 2016; Lebois et al., 2017).

*In vivo* calcium imaging allows for the observation of dynamic fluctuations in intracellular calcium levels in neurons, correlating with action potential generation and synaptic plasticity (Resendez and Stuber, 2015). This technique enables the simultaneous monitoring of neuronal activity across hundreds of cells (Fu et al., 2014; Stringer et al., 2019), facilitating long-term observations and the identification of spatial patterns among neurons with similar activity (Kerlin et al., 2010). While two-photon microscopy is a common method for *in vivo* calcium imaging (Denk et al., 1990; Ter Veer et al., 2017; Zatonyi et al., 2020), it typically requires immobilization and anesthesia, which can alter neuronal activity (Constantinople and Bruno, 2011; Xu et al., 2023). The development of compact single-photon fluorescence microscopes, or “miniscopes,” has addressed some of these challenges by enabling calcium imaging in freely moving animals (Flusberg et al., 2008; Ghosh et al., 2011; Jennings et al., 2015; Liberti et al., 2017; Jacob et al., 2018; Zhang et al., 2019; de Groot et al., 2020). This innovation has greatly advanced our understanding of neuronal dynamics and spatial encoding, particularly in neurodegeneration models such as 5xFAD mice (Gerasimov et al., 2020; Zhang et al., 2023), where progressive disruptions in calcium activity and spatial encoding correlate with neurodegenerative changes in the hippocampus (Zhang et al., 2023).

Electrophysiological recording provides direct measurements of neuronal activity with high temporal precision (Besnard et al., 2010; Davis et al., 2014; Donzis et al., 2018). A series of *in vivo* electrophysiological studies have been conducted on the 5xFAD transgenic mouse line. A deficit in gamma activity was observed in 5xFAD mice compared to wild-type (WT) mice during a behavioral test in virtual reality, which recorded local field potentials (LFP) from the CA1 area of the hippocampus. This deficit was observed at three months of age, prior to the formation of amyloid plaques or the onset of cognitive impairment (Iaccarino et al., 2016). Furthermore, *in vivo* recording of individual neurons in the CA1 region of 5xFAD mice revealed a deficit in synaptic connections and neuronal encoding, accompanied by significant disruptions in neuron activation during sharp waves and ripples (SWR), which constitute components of LFP (Prince et al., 2021). Moreover, in a discrete high-frequency range (65–110 Hz), a reduction in the amplitude of gamma oscillations (high-frequency LFP) in the left CA1 region of the hippocampus and an increase in the dentate gyrus (DG) were observed in 5xFAD mice compared to WT mice (Zhen et al., 2017). Additionally, analysis of LFP recordings from the dentate gyrus and perforant path of the hippocampus revealed disturbances in theta and gamma activity patterns in 5xFAD mice (Wang et al., 2020).

It is important to acknowledge the limitations of electrophysiological methods. For instance, they are less precise in identifying low-activity neurons (Wei et al., 2020) and have trouble keeping track of the same neuronal populations due to electrode encapsulation and movement (Tolias et al., 2007; Dhawale et al., 2017). Nevertheless, this method provides direct measurement of neuronal activity with high signal-to-noise ratio, temporal precision and dynamic range (Buzsaki, 2004). The simultaneous application of electrophysiological and *in vivo* imaging techniques may prove to be an effective approach to study complex neurobiological phenomena due to the acquisition of more neuronal activity data from a single experimental animal. Recent efforts have focused on integrating single-photon calcium imaging with electrophysiological recording to create more comprehensive tools for studying neuronal activity (Hur et al., 2024). Despite some advancements, challenges remain, such as the increased size and weight of integrated devices and the need for chronic fixation during long-term experiments (Wu et al., 2021; Kim et al., 2022; McCullough et al., 2022).

A review of the available scientific literature revealed no information on the *in vivo* electrophysiological state of the hippocampal alveus in 5xFAD mice during learning and memory formation. A single study was identified that performed *ex vivo* LFP recordings from the hippocampal alveus in 5xFAD mice, but the data from this study were not analyzed by the authors (Crouzin et al., 2013). To address this gap in knowledge, an analysis of hippocampal neuronal activity was performed in both wild-type (WT) and 5xFAD mice 5–6 months of age during a fear conditioning test. This study used an integrated approach, combining single-photon calcium imaging with wireless electrophysiological recording (Erofeev et al., 2023). The calcium imaging was focused on pyramidal neurons in the CA1 region of the hippocampus, while LFPs were simultaneously recorded in the hippocampal alveus at various stages of the behavioral test. It is anticipated that the incorporation of these sophisticated methodologies will facilitate the acquisition of novel insights into the neurodegenerative processes underlying Alzheimer’s disease, thereby further elucidating the mechanisms associated with this condition.

## 2 Materials and methods

### 2.1 Mice

Animals of both sexes were used in the study. B6SJL and 5xFAD mice were used for the experiments. 5xFAD mice (Jackson Laboratory, Bar Harbor, ME, USA; strain #034840-JAX) in a B6SJLF1 background were obtained from the Jackson Laboratory and used in experiments as follows: AAV injections were performed in 3-months-old mice, followed by implantation of a gradient refractive index (GRIN) lens integrated with a microelectrode at 4 months, and a fear conditioning test conducted at 5–6 months of age. The number of mice employed in the fear conditioning test (FCT) was distributed as follows: on the 1st and 2nd days of the test, the wild-type group comprised eight animals, while the 5xFAD group comprised nine animals. On the third day of the experiment, the number of animals in each group was six for the WT group and eight for the 5xFAD group. On the tenth day of the experiment, the number of mice in each group was six for the WT group and seven for the 5xFAD group. The decrease in the number of animals in each group was due to their exclusion from the experiment in case of implant loss or damage to the microelectrode cable.

Mice were housed at the Laboratory of Molecular Neurodegeneration of Peter the Great St. Petersburg Polytechnic University under standard conditions, including a 12:12-h light/dark cycle. Food and water were available *ad libitum*. All procedures adhered to the principles of the European Convention and the Declaration of Helsinki regarding the humane treatment of animals and were approved by the Bioethics Committee of Peter the Great St. Petersburg Polytechnic University in St. Petersburg.

### 2.2 Microelectrode

The designs of the microelectrode and the adapter cable were developed using Altium Designer software (Altium, San Diego, California, USA). The adapter cable was utilized to connect the microelectrode to the wireless electrophysiological module developed earlier by our team (Erofeev et al., 2023).

The microelectrode and adapter cable were fabricated from a polyimide substrate laminated with copper foil on both sides. In the first stage, a photosensitive material (photoresist) was applied to the blank in a clean room under yellow lighting to avoid ultraviolet light exposure. Next, the photoresist was exposed using a positive photomask corresponding to the microelectrode or adapter cable pattern. After removing the photomask, the image developed on the photoresist. The areas that weren’t exposed dissolved, but the areas that were exposed stayed on the blank because they polymerized and became insoluble. These areas were critical for selective copper electroplating. To protect the plated areas, a metal resist, which has a lower etching rate than copper, was applied. After removing the photoresist, the copper was etched, dissolving the unprotected copper and leaving a pattern of conductive tracks. The metal resist was then removed, and a polyimide cover layer with pre-formed holes corresponding to the contact locations was applied. In the final stage, the exposed copper areas were gold-plated to complete the microelectrode fabrication process.

Each side of the microelectrode featured 6 conductive channels, forming a total of 12 recording contacts. These contacts were arranged in a staggered pattern at the tip of the lower part of the microelectrode, which measured 5 × 1 mm. The microelectrode’s thickness was 0.2 mm. The dimensions of the recording contacts were 0.1 × 0.02 mm, with an inter-electrode distance of 0.3 mm. The microelectrode’s overall length was 20 mm, the polyimide ribbon cable’s length was 15 mm, and its width was 2 mm.

The microelectrode was connected to the wireless electrophysiological module via a Hirose BM10B (0.8)-20DP-0.4V connector (Hirose Electric, Kanagawa, Japan). The impedance of the microelectrode was assessed in a 0.1 M phosphate-buffered saline solution. Impedance measurements were conducted using an RLC meter (AMM-3035, AKTAKOM, Moscow, Russia). During the measurements, variable sinusoidal signals with a voltage amplitude of 5 mV and frequencies ranging from 0.1 to 1000 Hz were used. Within this frequency range, the impedance values varied from 6.28 to 0.14 MΩ

### 2.3 GRIN lens combined with a microelectrode

The microelectrode’s final form was created by positioning it around a GRIN lens with a diameter of 1.8 mm (0.25 pitch, #64-519, Edmund Optics, Florida, USA), ensuring that the ends of the lens and microelectrode were aligned. This assembly was secured using a metal ring fabricated from a 2 mm diameter steel tube that was longitudinally cut on one side. The structure was formed at a temperature of 300°C for 2 min. Subsequently, the microelectrode was attached to the GRIN lens using a gel-based cyanoacrylate adhesive, Loctite 454 (Henkel, Düsseldorf, Germany). The microelectrode flex cable was bent at a 90-degree angle relative to the GRIN lens, with a distance of 1.5 mm from the lens’s upper end to the bend point. This procedure ensured the lens was securely fixed during implantation into the laboratory animal’s brain, as well as the miniscope base plate’s subsequent fixation.

### 2.4 Stereotaxic surgical procedures

All surgical procedures were performed under anesthesia using isoflurane (induction at 4%, maintenance at 1%–2% after the animal was verified to have no pain reflexes).

#### 2.4.1 Stereotaxic AAV injections into the mouse hippocampus

For *in vivo* calcium imaging and simultaneous electrophysiological recording in the hippocampus of the right hemisphere, 3-months-old B6SJL and 5xFAD mice were injected with 1 μL of adeno-associated virus AAV5.Syn.GCaMP6f.WPRE.SV40 (#100837, Addgene) at a titer of 7 × 10^12^ viral particles per mL. Virus delivery to the hippocampus was performed at a rate of 100 nL per minute using a 75 RN syringe with a 5 μL capacity (#65460-03, Hamilton, Reno, Nevada, USA) according to the standard protocol (Cetin et al., 2006). The procedure was conducted using the following coordinates on a stereotaxic frame (68001, RWD Life Science, Shenzhen, China): AP −1.95; DV −1.5; ML −1.7, in mm from the bregma.

#### 2.4.2 Implantation of a GRIN lens combined with a microelectrode

Four weeks after virus injection, a GRIN lens with a diameter of 1.8 mm (0.25 pitch, #64-519, Edmund Optics, Florida, USA), combined with a microelectrode, was implanted. Relative to the viral injection coordinates, guide holes were drilled, and a circular craniotomy of 2.1 mm was performed using a dental burr. The bone fragment and dura mater were then carefully removed. In cases of bleeding, the implantation area was further washed with phosphate-buffered saline (PBS) and filled with a hemostatic sponge (FNPC Belkozin, Russia). On the side opposite to the craniotomy, a small hole was drilled using a dental burr to install an M1 × 3 micro screw, which was connected to a silver wire (#783500, AM-systems, Washington, USA). This wire was subsequently connected to the ground contact of the microelectrode.

To remove the cortical and corpus callosum layers above the hippocampus, a dental aspirator OM-1 (Utes, Russia) and a blunt-ended syringe were used (Zhang et al., 2019). During this procedure, the extraction area was periodically filled with PBS, and the process continued until a layer of vertical fibers of the hippocampal alveus appeared. Subsequently, the GRIN lens, combined with a microelectrode, was attached to the tip of the pipette holder using negative pressure generated by a dental aspirator. The pipette was then installed in the stereotaxic device and moved to the center of the craniotomy area. Afterward, the GRIN lens-microelectrode assembly was implanted to a depth of 1.4 mm (focal length of ∼250 μm for CA1 cells) relative to the skull surface, reaching the surface of the exposed tissues. The external surface of the GRIN lens and microelectrode was fixed to the skull using Cosmofen CA-500.200 cyanoacrylate glue (Weiss Chemie, Haiger, Germany). After the glue dried, the GRIN lens holder was removed, and the bone tissue was covered with light-curable dental cement (DentLight flow, VladMiVa, Russia). Upon completion of the surgery, isoflurane anesthesia was reduced to 0%, and the animal was subcutaneously injected with 0.1 mL of dexamethasone “Cortexon Retard” (SYVA Laboratorios S.A, Leon, Spain) before being transferred to a clean cage with a thermoregulated electric heating pad for recovery. In the initial postoperative period, the animal was provided with soft food, such as finely ground porridge.

#### 2.4.3 Baseplating

Three weeks following the GRIN lens implantation, the small aluminum base plate of the miniscope V3 was affixed to the animal’s skull over the previously applied dental cement. Using magnets and a locking screw, the base plate was attached to the bottom of the UCLA Miniscope v3 (Labmaker, Berlin, Germany). The miniscope was then connected to a data acquisition (DAQ) system (Labmaker, Berlin, Germany) and configured through the Portable Miniscope Data Acquisition software (Pomidaq, version 0.5.1^1^). Once the miniscope had been aligned with the implanted GRIN lens and a clear image of the fluorescent neurons had been obtained, the base plate was secured to the skull with dental cement.

### 2.5 Connecting the wireless module to the miniscope and data transmission

The wireless module was attached to the miniscope using electrically insulating double-sided adhesive tape. Power was supplied to the electrophysiological system’s wireless module using a 5 V voltage from the capacitor on the miniscope board, eliminating the need for an external battery.

The miniscope was connected to the data acquisition system using a CW2040-3650 SR coaxial cable (Cooner Wire, Chatsworth, California, USA). The DAQ, in turn, was connected to a personal computer via a USB 3.0 cable, which facilitated data transmission, power supply, and miniscope control. The DAQ received power from the USB port on the personal computer, which was then transmitted to the miniscope via the coaxial cable. Consequently, a 5 V voltage was generated across the miniscope’s printed circuit board (PCB) capacitor, which then powered the wireless electrophysiological module.

Data transmission from the wireless module to the personal computer was carried out using the Bluetooth Low Energy protocol. The wireless module supported various sampling rates: 1000 Hz for two channels, 500 Hz for four channels, 250 Hz for eight channels, 125 Hz for sixteen channels, and 62 Hz for thirty-two channels.

### 2.6 Fear conditioning test

A fear conditioning test was conducted over three consecutive days a week after the baseplating procedure, culminating on the tenth day after the start of the experiment. The experiments were performed using a standard conditioning chamber (25 cm × 20 cm × 30 cm) with a stainless-steel floor connected to an electric shock generator. The chamber was housed in a soundproof isolation cubicle. Measurements were accomplished through two cameras (Web camera Logitech C920 and Logitech C300, Logitech, Switzerland), connected to a computer with video freeze software (AUTO_URAI_4 developer Dr. V.V. Sizov, Institute of Experimental Medicine, Saint Petersburg, Russia, I.P. Pavlov Department of Physiology, sizoff@list.ru).

To exclude potential freezing behavior induced by the novel environment, the mice were habituated to the chamber. On the first day (habituation), the mice were brought into the habituation room and individually placed into the training chamber. The animals freely explored the new environment without exposure to a foot shock (unconditioned stimulus, US) or a tone (conditioned stimulus, CS). This phase lasted for 300 s, during which all external conditions (lighting, sound, smell, the positioning of the chamber, and the surrounding environment) remained constant. After the 300-s period, the animals were returned to their home cages.

On the second day (24 h later), each mouse was placed in the conditioning chamber for 120 s without exposure to foot shock or tone (baseline). Subsequently, the mice received two repetitions of a 20-s tone (5000 Hz, 55 dB), each simultaneously ending with a 1.5-s foot shock (0.5 mA) (trial 1 and trial 2). A 120-s recovery period was provided between trial 1 and trial 2. After the second repetition, the mice were allowed to recover for 60 s before being returned to their home cages.

On the third day (24 h later), the mice were placed back in the familiar fear conditioning chamber without exposure to tones or foot shocks. In order to assess the retrieval of contextual memory, the freezing behavior was recorded over a 180-s period. In the tone fear retrieval trial, conducted 1 hour after the contextual test, the mice were placed in an altered conditioning chamber. The chamber was distinguished by the following characteristics: black and white striped walls, a covered floor, additional light and a vanilla extract scent. After 180 s of baseline recording in the altered conditioning chamber (pre-tone), a tone (5000 Hz, 55 dB) was presented for 180 s to assess the response to the conditioned signal in a new environment (tone test). The freezing behavior before and during the tone was measured. The mice were then given a 60-s rest before being returned to their home cages. On the tenth day, the behavioral test was repeated in the same manner as on the third day.

Before the beginning of each stage of the fear conditioning tests, a miniature fluorescent microscope was mounted on the baseplate on the mouse’s head. The implanted microelectrode was also connected to the wireless electrophysiological module via an adapter cable. At the end of each stage, the miniscope and microelectrode were disconnected before returning the mouse to its home cage.

The equipment for the fear-conditioning test was connected to the Miniscope DAQ’s output toggle signal for synchronization. Synchronization of electrophysiological recording with the miniscope and video cameras was performed using Syntalos software, for timestamp-synchronized parallel data acquisition.^2^ The experimental programs were launched simultaneously using open-source software AutoHotkey v2.0.^3^

### 2.7 *In vivo* calcium imaging and electrophysiology recordings

Calcium imaging and electrophysiological recording of neuronal activity were conducted inside a soundproof, insulated cabin for the fear conditioning test, which was lined with a thick layer of grounded aluminum foil. This setup was designed to shield the internal environment from external electromagnetic radiation. Neuronal activity was recorded during the fear conditioning test on days 2, 3, and 10.

#### 2.7.1 Calcium imaging with miniscope V3

Eight weeks following the stereotaxic injection of AAV, calcium imaging of hippocampal neurons expressing GCaMP6f in the CA1 region was conducted using a UCLA Miniscope v3, which weighs 2.8 g. The implanted gradient lens was utilized to record the fluorescence of hippocampal neurons under blue light excitation. The light source was a 470 nm blue LED, LXML-PB01-0030 (Lumileds, Haarlemmermeer, Netherlands), housed within the UCLA Miniscope v3. The focus of the fluorescent neuron images was adjusted using the miniscope’s focusing slider. The process of fluorescence imaging of hippocampal CA1 neurons using the miniscope is comprised of a number of distinct stages. In the initial stage, the blue LED light is collimated and focused by a N-BK7 half-spherical lens (Edmund Optics, Barrington, New Jersey, USA). Subsequently, the light is filtered through an ET470/40x excitation filter (Chroma Technology Corp., Bellows Falls, Vermont, USA) in order to emit light. At 470 nm ± 20 nm, the light is reflected by a T495lpxr dichroic mirror (Chroma Technology Corp., Bellows Falls, Vermont, USA), and directed through a gradient lens to focus on hippocampal neurons.

Subsequently, the emitted fluorescence from neurons passes back through the gradient lens, reflecting off the dichroic mirror, and passing through an ET525/50m emission filter (Chroma Technology Corp., Bellows Falls, Vermont, USA), which transmits light at 525 nm ± 25 nm. This light is corrected with an achromatic doublet lens N-BAF10/N-SF10 (Edmund Optics, Barrington, New Jersey, USA) in order to minimize chromatic aberrations and focus on the CMOS sensor.

The light signal is then converted into electrical signals on the CMOS sensor, which are transmitted via a flexible coaxial cable CW2040-3650 SR (Cooner Wire, Chatsworth, California, USA) to a data acquisition controller system (Labmaker, Berlin, Germany). Finally, the data from the DAQ system is transferred to a personal computer via a USB 3.0 cable using the USB Video Class (UVC) protocol for further analysis and visualization.

*In vivo* calcium imaging data were acquired using the open-source software Pomidaq v0.5.1.^1^ Video recordings were captured at a rate of 20 frames per second. The gain level of the miniscope’s CMOS sensor was set to a value of “medium.” The intensity of the LXML-PB01-0030 LED was adjusted to range from 8% to 16% of its maximum power (35 lumens). All data was saved in AVI format with a resolution of 752 × 480 pixels.

#### 2.7.2 Electrophysiology recordings with wireless electrophysiological system

Neuronal activity was recorded using a wireless electrophysiology system weighing 0.95 g (Erofeev et al., 2023). The system consisted of a wearable wireless module and open-source software for data management and recording. The wearable module was based on the NRF52805-CAAA-R7 microprocessor-transceiver (Nordic Semiconductor, Trondheim, Norway) and a 32-channel unipolar input amplifier chip RHD2132 (Intan Technologies, Los Angeles, California, USA). The RHD2132 chip provided a differential gain of 192 V/V. The key components of the wearable module included two 20-position board-to-board connectors BM10NB(0.8)-20DS-0.4V(51) (Hirose Electric, Kanagawa, Japan); ceramic antenna 2450AT18A100E (Johanson Technology, Camarillo, California, USA); lithium-ion and lithium-polymer battery charge controller MCP73812T-420I/OT (Microchip Technology Inc., Chandler, Arizona, USA); low dropout voltage regulator MCP1700-3002E/TO (Microchip Technology Inc., Chandler, Arizona, USA); voltage regulator TLV70033_SOT23-5 (Texas Instruments, Dallas, Texas, USA); Hall sensor KTH1601SL-ST3 (CONNTEK Microelectronics Technology, Xuanzhou, China); LED KPHHS-1005SECK (Kingbright, Shanghai, China); and 32 MHz quartz resonator NX2520SA (NDK, Tokyo, Japan).

Electrophysiological data were recorded using the open-source software ble_mouse.py, designed for wireless electrophysiological systems and available at https://github.com/lmn-projects/WES-2.0/tree/main/software. The neuronal activity was recorded in a two-channel mode without the application of noise filtering at a sampling rate of 1000 Hz. The registration process was conducted in dual-channel mode as the current firmware version is only compatible with a 1000 Hz sampling rate when utilizing two channels. The data were saved in CSV format, a text-based format commonly used for storing tabular data.

### 2.8 Slice preparation

After the fear conditioning test, brain tissue preparation and fixation were conducted to assess the viral transduction in the mouse hippocampus. The mice were initially anaesthetized using a urethane solution (250 mg/mL in 0.9% NaCl, Sigma-Aldrich, St. Louis, MO, USA), administered via intraperitoneal injection. The intracardiac perfusion was conducted using an ice-cold 1.5% paraformaldehyde (PFA, Sigma-Aldrich, USA) solution in phosphate buffer (pH 7.4, Sigma-Aldrich, USA), with a total volume of 30 ml administered over a period of 180 s. Subsequently, the brains were extracted and post-fixed in 4% PFA at +4°C for 1 week. Thin hippocampal slices (50 μm) from the fixed brains were prepared using a microtome (5100 MZ, Campden Instruments, United Kingdom) and stored in sucrose +0.8% PFA solution at 4°C. The slices were then rinsed in PBS and placed in a 24-well plate containing 0.5% PFA for storage until further processing. Finally, the slices were mounted on slides using Aqua Poly/Mount (Polysciences, Inc. #18606) and imaged with a confocal microscope (ThorLabs, Newton, NJ, USA) to analyze the tissue.

### 2.9 Quantification and statistical analysis

Data recorded with the Miniscope and wireless electrophysiology system were processed using the Anaconda distribution (version 2024.02-1) of the Python and R programming languages for scientific computing.^4^

#### 2.9.1 Freezing analysis

Behavior video data were processed using the “POLE_KREST” software (developer Dr. V.V. Sizov, Institute of Experimental Medicine, Saint Petersburg, Russia, I.P. Pavlov Department of Physiology, sizoff@list.ru), which calculated the duration of freezing episodes in seconds. Subsequently, these values were converted into percentages of the total duration of the behavioral test stage.

#### 2.9.2 Miniscope data analysis

The video data from the miniscope was preprocessed in the Jupyter Notebook environment using the open-source package Minian^5^ (Dong et al., 2022). Default parameters were utilized during the analysis. The installation of the Minian package was conducted using the package manager Mamba.^6^ The processing of raw data with the Minian (Dong et al., 2022) package enabled the determination of spontaneous Ca^2+^ activity in neurons, expressed as relative changes in fluorescence intensity (*△F/F*). Quantitative analysis of the preprocessed data was performed using the open-source package NeuroActivityToolkit^7^ (Gerasimov et al., 2023). The active phase of the signal was isolated by the “*spike*” method, in which only the increasing part of the fluorescence intensity of the calcium indicator was included in the active state of the neuron. The parameters of this process are as follows: cold – 4, warm – 5, window – 5.

The results of the PSTH calcium activity analysis are presented as the median value, with the median absolute deviation. The PSTH was visualized using the Matplotlib library, which is part of the Jupyter Notebook.

Calcium activity at specific stages of the FCT was assessed using the following parameters: burst rate, network spike rate (NSR), network spike duration (NSD), and network spike peak (NSP). The parameter “burst rate” was used to assess the average number of calcium events per neuron across the entire network per minute. Burst rate was calculated as follows:


$$B\,u\,r\,s\,t\,r\,a\,t\,e=\frac{\sum_{x\in A}a\,c\,t\,i\,v\,e\,\left(x\right)}{t},$$

where *A* represents the set of neurons, *active(x)* indicates whether a neuron is active (1 if active, 0 otherwise), and *t* denotes the time interval (1 min).

The network spike rate parameter was employed to evaluate the proportion of active neurons within 1 s for the corresponding interval of the fear conditioning test. NSR was calculated using the following formula:


$$N\,e\,t\,w\,o\,r\,k\,s\,p\,i\,k\,e\,r\,a\,t\,e=\frac{\sum_{x\in A}a\,c\,t\,i\,v\,e\,\left(x\right)}{s\,i\,z\,e\,\left(A\right)},$$

where *A* denotes the set of neurons, *active(x)* equals 1 if the neuron is active and 0 otherwise, and *size(A)* represents the number of neurons in the set.

The network spike duration parameter characterizes the period during which the number of simultaneously active neurons exceeds a specified threshold. NSD was calculated as follows:


$$N\,e\,t\,w\,o\,r\,k\,s\,p\,i\,k\,e\,d\,u\,r\,a\,t\,i\,o\,n=\frac{\sum_{i=1}^{T}\frac{\sum_{x\in A}a\,c\,t\,i\,v\,e\,\left(x_{i}\right)}{s\,i\,z\,e\,\left(A\right)}>t\,h\,r\,e\,s\,h\,o\,l\,d}{T},$$

where *active(x_*i*_)* equals 1 if this neuron is active, and 0 otherwise at the *i*-th moment of time, threshold is the established threshold value (0.8), and *T* is the time interval of the whole recording corresponding to the stage of the fear conditioning test.

The network spike peak represents the maximum number of neurons that are simultaneously active during a recording period within a specified time interval. NSP was calculated using the following formula:


$$N\,e\,t\,w\,o\,r\,k\,s\,p\,i\,k\,e\,p\,e\,a\,k=m\,a\,x_{i=1}^{t}\,\left(\frac{\sum_{x\in A}a\,c\,t\,i\,v\,e\,\left(x_{i}\right)}{s\,i\,z\,e\,\left(A\right)}\right),$$

where *size(A)* represents the number of neurons in the set, *active(x_*i*_)* indicates the active state (1 if the neuron is active) at time *i*, and *t* denotes the time interval (1 s).

#### 2.9.3 LFP analysis

The analysis of electrophysiological data was conducted in the Jupyter Notebook environment. Data was imported from CSV files utilizing the .genfromtxt function provided by the NumPy library. Baseline alignment of neuronal activity recordings was achieved by applying the median filter (medfilt), also available within the NumPy library. To suppress electrical noise, a 5th-order lowpass Butterworth filter with a cutoff frequency of 50 Hz was implemented using the signal.butter and signal.filtfilt functions from the SciPy library. The amplitude of local field potentials was determined through peak detection using the signal.find_peaks function, with the detection threshold set at 0.1 mV. In addition, power spectral analysis of LFPs was performed on raw data using the Welch method, implemented with the Welch function from the SciPy library. The analysis utilized a segment duration of 10 s (window_duration), corresponding to nperseg = fs * window_duration data points, where fs is the sampling frequency. The resulting power spectra were log-transformed (np.log10) to enhance visualization. The resulting plots emphasized the frequency bands of interest, including Delta (0.5–4 Hz), Theta (4–8 Hz), Alpha (8–13 Hz), Beta (13–30 Hz), and Gamma (30–100 Hz), to facilitate comparative analysis. Data smoothing was performed using the Gaussian filter (gaussian_filter1d) to calculate the smoothed mean and standard error of the mean. Data visualization was performed using the Matplotlib library.

The signal-to-noise ratio (SNR) was determined using the following formula:


$$S\,N\,R=20\,l\,o\,g_{10}\,\left(\frac{A_{s\,i\,g\,n\,a\,l}}{A_{n\,o\,i\,s\,e}}\right),$$

where *A*_*signal*_ – LFP amplitude, a *A*_*noise*_ – noise amplitude.

The signal power (*P*) and its root mean square value (*V*_*rms*_) were calculated using the following formulas:


$$P=\frac{1}{N}\,\sum_{i=1}^{N}x_{i}^{2},$$

$$V_{r\,m\,s}=\sqrt{\frac{1}{N}\,\sum_{i=1}^{N}x_{i}^{2}},$$

where x_*i*_ represents the amplitude of the i-th local field potential, and *N* is the total number of local field potentials.

The mean SNR values for the WT mouse group were 16 decibels (dB) and 15 dB for the 5xFAD mouse group, respectively. The mean *P* were 0.03 ± 0.005 W for wild-type mice and 0.02 ± 0.005 W for Alzheimer’s disease mice. The *V*_*rms*_ were 0.18 mV and 0.16 mV, respectively.

### 2.10 Statistical analysis

The data are presented as the mean ± standard error of the mean (SEM). Statistical analyses were conducted using GraphPad Prism version 8.0.1 (GraphPad Software, Boston, MA, USA). The normality of sample distributions was evaluated using the Shapiro-Wilk test, homogeneity was evaluated using the Bartlett’s test and variances were compared using the *F*-test or Levene’s test. The outliers were identified using Grubbs’ test (alpha = 0.05), values that were most distant from the others but not significant outliers were not excluded. Data were analyzed using the *t*-test, Welch’s *t*-test, one-way ANOVA with a *post hoc* Tukey’s test, Kruskal–Wallis test with a *post hoc* Dunn’s test or Mann-Whitney test.

To calculate the difference (Δ) in freezing behavior between WT and 5xFAD mice on days 3 and 10 during the tone test (Δ_3_^tone^ and Δ_10_^tone^) and the contextual test (Δ_3_^context^ and Δ_10_^context^), the mean freezing duration and the standard error of the mean were computed for each group using GraphPad Prism software. Subsequently, Δ_3_^tone^, Δ_10_^tone^, Δ_3_^context И^ Δ_10_^context^ were determined as the difference (Δ) between the mean freezing values of WT and 5xFAD mice for the corresponding day and test. A paired *t*-test was then performed to compare Δ_3_^tone^ with Δ_10_^tone^ and Δ_3_^context^ with Δ_10_^context^.

To ascertain the correlation between the physiological and behavioral data for both the WT and 5xFAD mouse groups, the mean values of the corresponding parameters were calculated for each stage of the fear-conditioning test. These included the number of freezing, the electrophysiological values of the amplitude and frequency of local field potentials, and the calcium imaging parameters: burst rate, network spike rate, network spike peak, and network spike duration. Furthermore, a correlation analysis was conducted between the number of freezing episodes and each of the physiological parameters in the GraphPad Prism software, resulting in the corresponding Pearson correlation coefficients (*r*).

## 3 Results

**FIGURE 1:**
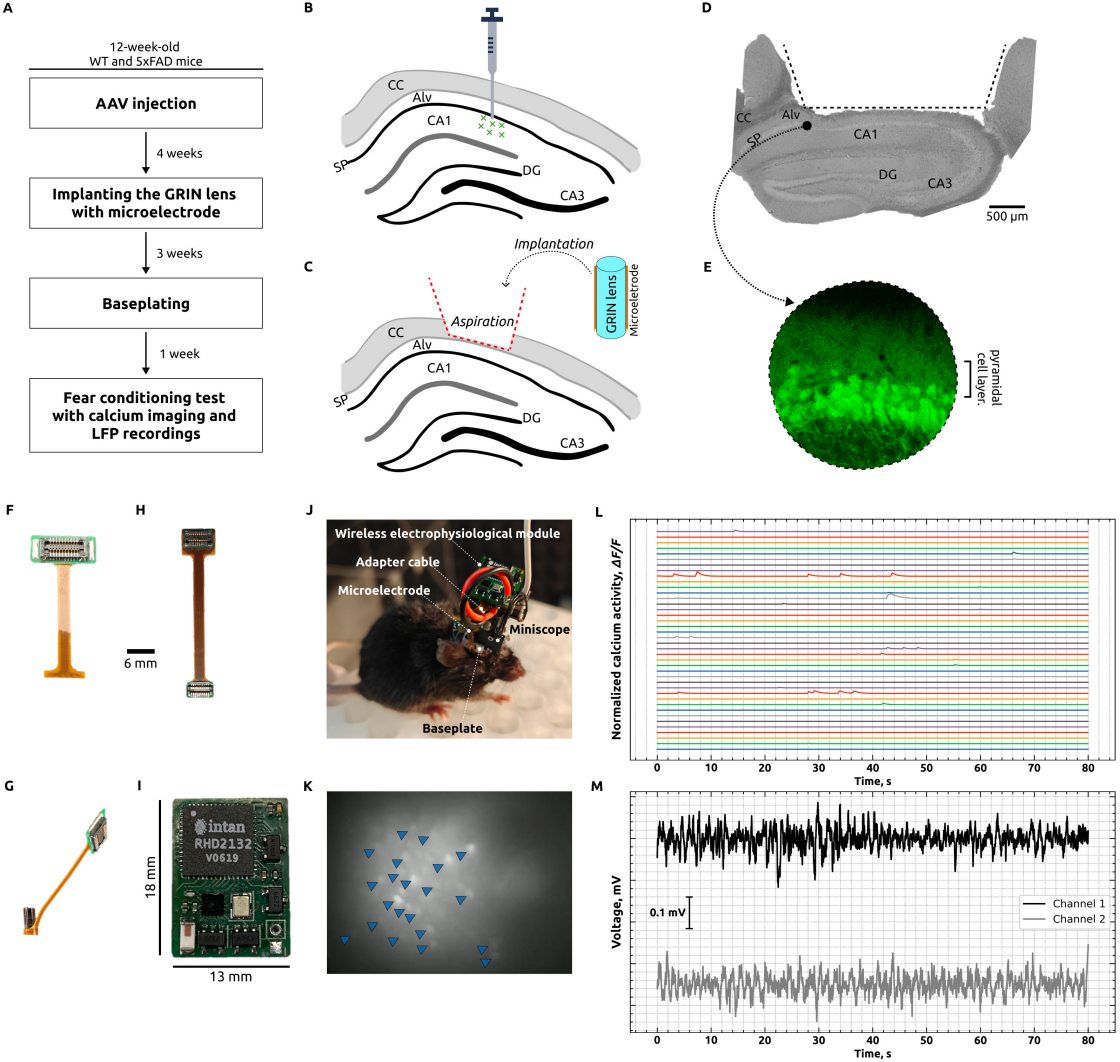
Simultaneous calcium imaging and *in vivo* registration of local field potentials. **(A)** Primary phases of experimentation: AAV injections were administered unilaterally into the hippocampus of 12-weeks-old wild-type (WT) and Alzheimer’s disease (AD) mice. Subsequently, a GRIN lens with a microelectrode was implanted after a period of 4 weeks. Following a 3-weeks period, a baseplate was affixed, and 1 week later, a fear conditioning test was conducted with simultaneous calcium imaging of CA1 neurons and LFP recording from the hippocampal alveus. **(B)** Diagrammatic representation depicts the injection of AAV5.Syn.GCaMP6f.WPRE.SV40 in the vicinity of the CA1 cell layer. CC, corpus callosum; Alv, hippocampal alveus; SP, stratum pyramidale; DG, dentate gyrus; CA1 and CA3, hippocampal regions. **(C)** Diagrammatic representation of the process of brain tissue aspiration, including the complete removal of the corpus callosum, followed by the implantation of a GRIN lens with a microelectrode. **(D)** A photograph depicts a 4% PFA-fixed hippocampal slice, taken post-experiment, which illustrates the region that was aspirated and outlined for lens and microelectrode implantation. **(E)** Visualization of GCaMP expression in the hippocampus by confocal microscopy. **(F)** Polyimide microelectrode. **(G)** GRIN lens with microelectrode. **(H)** Adapter cable for the purpose of connecting the microelectrode to the wireless electrophysiological module. **(I)** A wireless electrophysiological module. **(J)** Photograph of a mouse with a miniscope and wireless electrophysiology module, which enables the concurrent acquisition of calcium imaging and electrophysiological data. **(K)** The unprocessed fluorescent image of neurons was captured using a miniscope. The image was generated by overlaying frames collected over a 5-s period. Individual fluorescent neurons are indicated by blue triangles. **(L)** Normalized spontaneous Ca^2 +^ activity following processing in Minian. All traces represent *△F/F* of individual neurons. **(M)** Exemplification of an electrophysiological recording conducted concurrently with calcium imaging, utilizing a wireless system.

In this study, we performed *in vivo* one-photon calcium imaging of CA1 hippocampal neurons concurrently with wireless electrophysiological recordings from the hippocampal alveus in freely moving mice to investigate neural alterations during a fear conditioning test. The sequential experimental design employed to achieve these simultaneous recordings is schematically summarized in Figure 1A. Adult B6SJL mice (wild-type, WT) and mice modeling Alzheimer’s disease (5xFAD) received unilateral hippocampal injections of the adeno-associated virus AAV5.Syn.GCaMP6f.WPRE.SV40 to target CA1 pyramidal neurons (Figure 1B). Four weeks post-injection (Figure 1C), the animals were implanted with a GRIN lens combined with a microelectrode (Figure 1G). The region that was aspirated and delineated for the implantation of the lens–microelectrode assembly is illustrated in Figure 1D, and GCaMP6f expression was confirmed by fluorescence imaging (Figure 1E). Three weeks later, a baseplate was affixed to the animal’s skull to secure the miniscope and its associated wireless module (Figure 1J). One week thereafter, a fear conditioning test was performed during which simultaneous calcium imaging of CA1 hippocampal neurons (Figures 1K, L) and local field potential recording from the hippocampal alveus (Figure 1M) were conducted using the microelectrode (Figure 1F), which was connected to a wireless electrophysiology module (Figure 1I) via an adapter cable (Figure 1H).

### 3.1 Fear conditioning test

**FIGURE 2:**
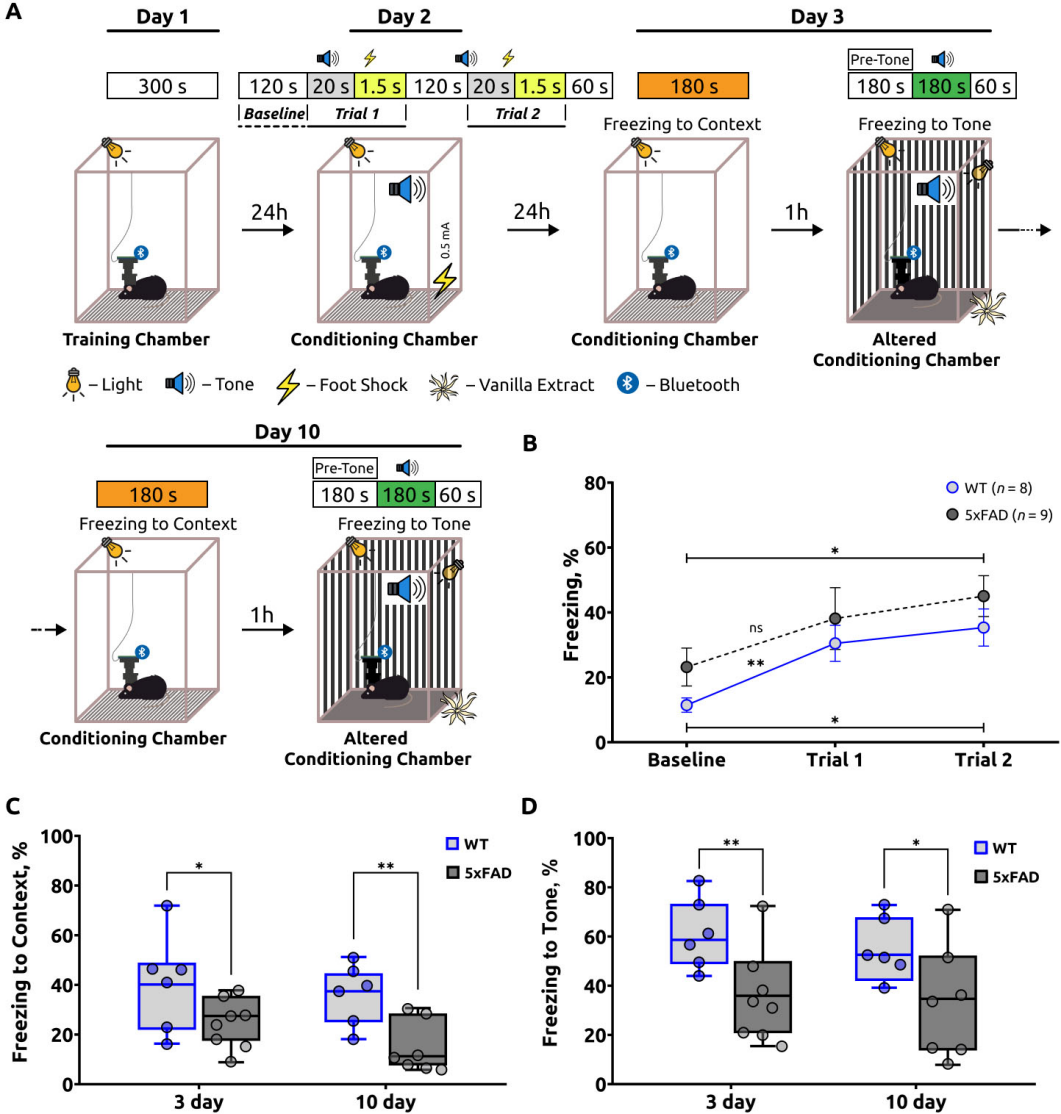
A summary of the freezing behavior observed in the fear conditioning test (FCT). **(A)** Schematic of the fear-conditioning protocol. **(B)** The freezing percentage during the acquisition stage of the FCT. The freezing percentage increased in both groups during the two trials, indicating that the mice were learning. An unpaired *t*-test was used to compare Baseline and Trial 1, as well as Baseline and Trial 2 within the WT and within the 5xFAD groups. For the WT group, Welch’s *t*-test was applied to compare Baseline and Trial 1. Statistical significance is indicated as *p < 0.05, **p < 0.01, ns: *p* > 0.05. The data are presented as the mean ± SEM. **(C)** Freezing response to the unconditioned room contextual test (Context). On days 3 and 10 of the FCT, the percentage of freezing was significantly lower in 5xFAD mice than in WT mice. An unpaired *t*-test was employed for day 3, and a Mann-Whitney test for day 10, with statistical significance denoted as *: *p* < 0.05, **: *p* < 0.01. **(D)** The freezing response to the conditioned stimulus tone test (Tone). The percentage of freezing in this test was also significantly lower in 5xFAD mice compared to the control group of mice on days 3 and 10 of the FCT. An unpaired *t*-test was employed for both days 3 and 10, with statistical significance denoted as *: *p* < 0.05, **: *p* < 0.01. The data are presented as box-and-whisker plots: the line within the box represents the median, the box boundaries indicate the 25th and 75th percentiles, and whiskers show the minimum and maximum values (excluding outliers).

On the second day of the fear conditioning test (FCT), the animals (WT: *n* = 8, 5xFAD: *n* = 9) were placed in the conditioning chamber for a period of 120 s in order to assess their baseline fear levels (Baseline) and freezing behaviors during the training sessions (trial 1 and trial 2). On this day of the FCT (Figure 2A), the baseline freezing level was analyzed and expressed as a percentage of the test’s phase duration. Furthermore, the freezing behaviors were assessed during the training sessions that involved exposure to the unconditioned stimulus (US) and the conditioned stimulus (CS) (trials 1 and 2) (Figure 2B). The baseline freezing percentage in 5xFAD mice was 23.17 ± 5.84 and in WT mice was 11.43 ± 2.19. In trial 1, freezing increased significantly (*p* = 0.0081) in wild-type mice (30.45 ± 5.56), indicating that the mice were learning, but there was no significant increase (*p* = 0.1960) in freezing in 5xFAD mice (38.11 ± 9.51). In trial 2, mean percent freezing increased significantly from baseline in both 5xFAD (45.03 ± 6.30; *p* = 0.0316) and WT (35.34 ± 5.73; *p* = 0.0111) mice, indicating that the mice were learning (Chen et al., 2012).

On the third and tenth days of the FCT, an investigation was conducted to ascertain the relationship between fear (freezing behavior) and the specific spatial context (unconditioned room context test, contextual test). In the conditioning chamber, the freezing response was employed as an indicator of hippocampal-dependent contextual memory. A notable decline in the freezing response was observed in the 5xFAD mice on both day 3 (25.67 ± 2.93) and day 10 (15.92 ± 3.17). In comparison to WT mice, who demonstrated heightened freezing behavior on days 3 and 10 (38.69 ± 5.57 and 34.89 ± 3.76), respectively (Figure 2C). The statistical analyses indicated significant differences with *p*-values of 0.0474 for day 3 and 0.0076 for day 10. These findings indicate that hippocampal-dependent contextual memory is impaired in 5xFAD mice in comparison to WT mice (Zernov et al., 2022).

On the third and tenth days, 1 h after the unconditioned room context test, the animals were placed in an altered conditioning chamber, in accordance with the experimental design. The chamber had been modified to alter the walls, floor, lighting, and odors (vanilla extract). This procedure was designed with the objective of evaluating the behavioral response of the animals to the novel environment (pre-tone) and conditioned signal (tone) in an environment that was previously unknown to them. The results obtained from the altered conditioning chamber were found to be comparable to those observed in the contextual test (Figure 2D). Following the CS period (tone test), mice with 5xFAD mice exhibited a reduced freezing response on both day 3 (38.32 ± 5.64) and day 10 (35.00 ± 6.40) of the test. This indicates a reduction in the strength of the learned association in the Alzheimer’s disease mice, as evidenced by their lower freezing response in comparison to the wild-type mice. The WT mice exhibited a freezing percentage of 60.25 ± 4.02 on day 3 and 55.19 ± 4.57 on day 10, respectively (Figure 2D).

To further analyze of the differences between WT and AD mice, the differences (Δ) in freezing behavior between the two groups were calculated for days 3 and 10 during the tone test (Δ_3_^tone^ and Δ_10_^tone^) and the contextual test (Δ_3_^context^ and Δ_10_^context^). In the tone test, the difference between WT and 5xFAD mice was Δ_3_^tone^ = 0.227 ± 0.036 on day 3 and Δ_10_^tone^ = 0.196 ± 0.068 on day 10. A paired *t*-test was conducted to compare Δ_3_^tone^ and Δ_10_^tone^, and no statistically significant differences were identified (*p* = 0.492). These findings indicate a notable reduction in freezing behavior in 5xFAD mice compared to WT mice on both day 3 and day 10 in the tone test.

In the contextual test, the differences between WT and 5xFAD mice were Δ_3_^context^ = 0.126 ± 0.064 and Δ_10_^context^ = 0.175 ± 0.061. Similarly, a paired *t*-test was employed to compare Δ_3_^context^ and Δ_10_^context^, which also demonstrated no statistically significant differences (*p* = 0.271). This suggests a consistently reduced percentage of freezing behavior in 5xFAD mice on both day 3 and day 10 during the contextual test.

### 3.2 Miniscope calcium imaging of CA1 hippocampal pyramidal neurons

**FIGURE 3:**
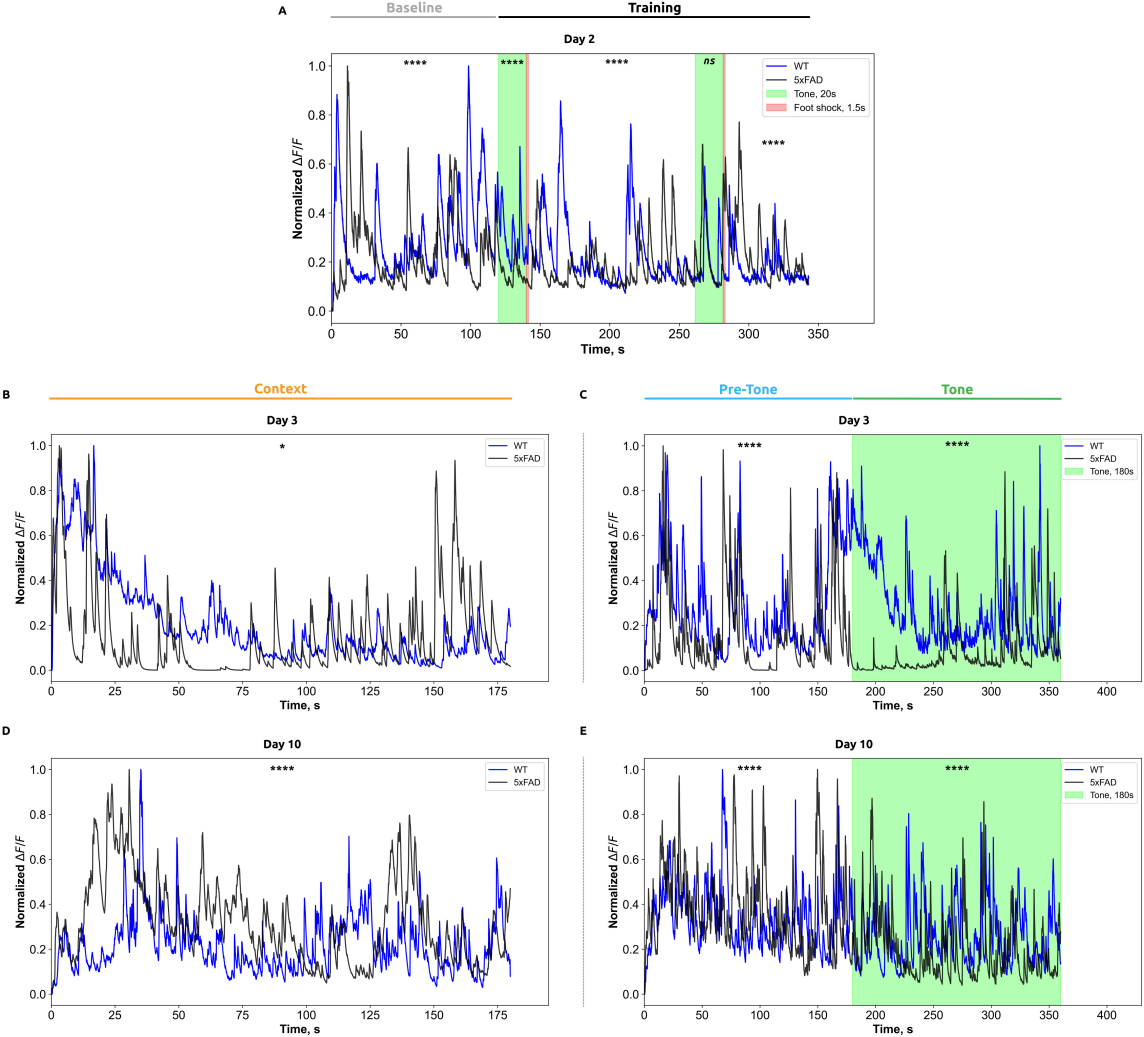
Normalized calcium activity (*ΔF/F*) in CA1 hippocampal neurons is presented for each phase of the fear-conditioning test: **(A)** Day 2 of testing; **(B,C)** Day 3; and **(D,E)** Day 10, recorded in the standard and altered chambers, respectively. The *x*-axis indicates time (in seconds), while the *y*-axis represents changes in normalized *ΔF/F* fluorescence. Data from wild-type mice are shown in blue, and those from Alzheimer’s disease model mice are shown in black. Stimulus presentation periods are marked by color: the auditory tone is indicated in green, and the foot shock is represented by a red vertical line. For analysis, calcium activity traces were segmented according to the defined phases of the fear-conditioning test; within each phase, the data were binned at 0.05-s intervals. For each interval, mean *ΔF/F* values were calculated for each animal, then averaged across all animals within each group (WT and 5xFAD). Data are presented as group means. Statistical analysis was performed using the Mann–Whitney test, with significance levels indicated as follows: *p < 0.05; ****p < 0.0001.

During the fear conditioning test, calcium imaging of pyramidal neurons from the CA1 region (stratum pyramidale) of the hippocampus was conducted using a miniscope. The data were initially processed using Minian to derive information on calcium activity, which was represented as *ΔF/F*. The findings are summarized in Figure 3.

On the second day of the FCT, during the baseline period (Figure 3A), before any auditory cue (Tone, CS) or foot shock (US), WT mice exhibited significantly higher median *ΔF/F* values (0.27 ± 0.10) compared to 5xFAD mice (0.23 ± 0.09; Mann-Whitney test, *p* < 0.0001). During the initial CS (trial 1), WT mice demonstrated a substantial increase in *ΔF/F* (0.31 ± 0.07) compared to 5xFAD mice (0.16 ± 0.03; Mann-Whitney test, *p* < 0.0001). Following the initial US (post-shock period), WT mice exhibited an increase in *ΔF/F* (0.19 ± 0.05) compared to AD mice (0.16 ± 0.04; Mann-Whitney test, *p* < 0.0001). In response to the second CS, no significant differences in *ΔF/F* were observed between WT mice (0.17 ± 0.05) and 5xFAD mice (0.19 ± 0.06; Mann-Whitney test, *p* = 4817). In contrast, in the posts-hock period after the second US, *ΔF/F* values were significantly different between the groups. 5xFAD mice exhibited significantly higher *ΔF/F* values (0.21 ± 0.06) compared to WT mice (0.16 ± 0.02; Mann-Whitney test, *p* < 0.0001).

On the third day of the contextual test (Figure 3B), 5xFAD mice demonstrated significantly higher initial *ΔF/F* activity (0.17 ± 0.14) compared to WT mice (0.15 ± 0.08; Mann-Whitney test, *p* = 0.0118).

On the third day of the tone test (Figure 3C) before the CS (0–180 s), WT mice demonstrated a significantly higher median change in the ratio of fluorescence of 0.23 ± 0.11 compared to 5xFAD mice (0.20 ± 0.16; Mann-Whitney test, *p* < 0.0001). During the tone-stimulation period (180–360 s), WT mice demonstrated the ratio of fluorescence of (0.20 ± 0.08), while 5xFAD mice exhibited significantly reduced *ΔF/F* values (0.06 ± 0.04; Mann-Whitney test, *p* < 0.0001) compared to WT group.

On the tenth day of the contextual test (Figure 3D), wild-type (WT) mice demonstrated significantly higher *ΔF/F* values (0.26 ± 0.10) in comparison to 5xFAD mice (0.17 ± 0.07; Mann-Whitney test, *p* < 0.0001). On the tenth day of the tone test (Figure 3E) before the CS (0–180 s), the *ΔF/F* values were: 0.28 ± 0.08 for WT mice and 0.26 ± 0.08 for 5xFAD mice (Mann-Whitney test, *p* < 0.0001). During the tonal stimulation period (180–360 s), *ΔF/F* in WT mice was 0.20 ± 0.07, whereas in 5xFAD mice, the values were significantly lower at 0.11 ± 0.05 (Mann–Whitney test, *p* < 0.0001).

**FIGURE 4:**
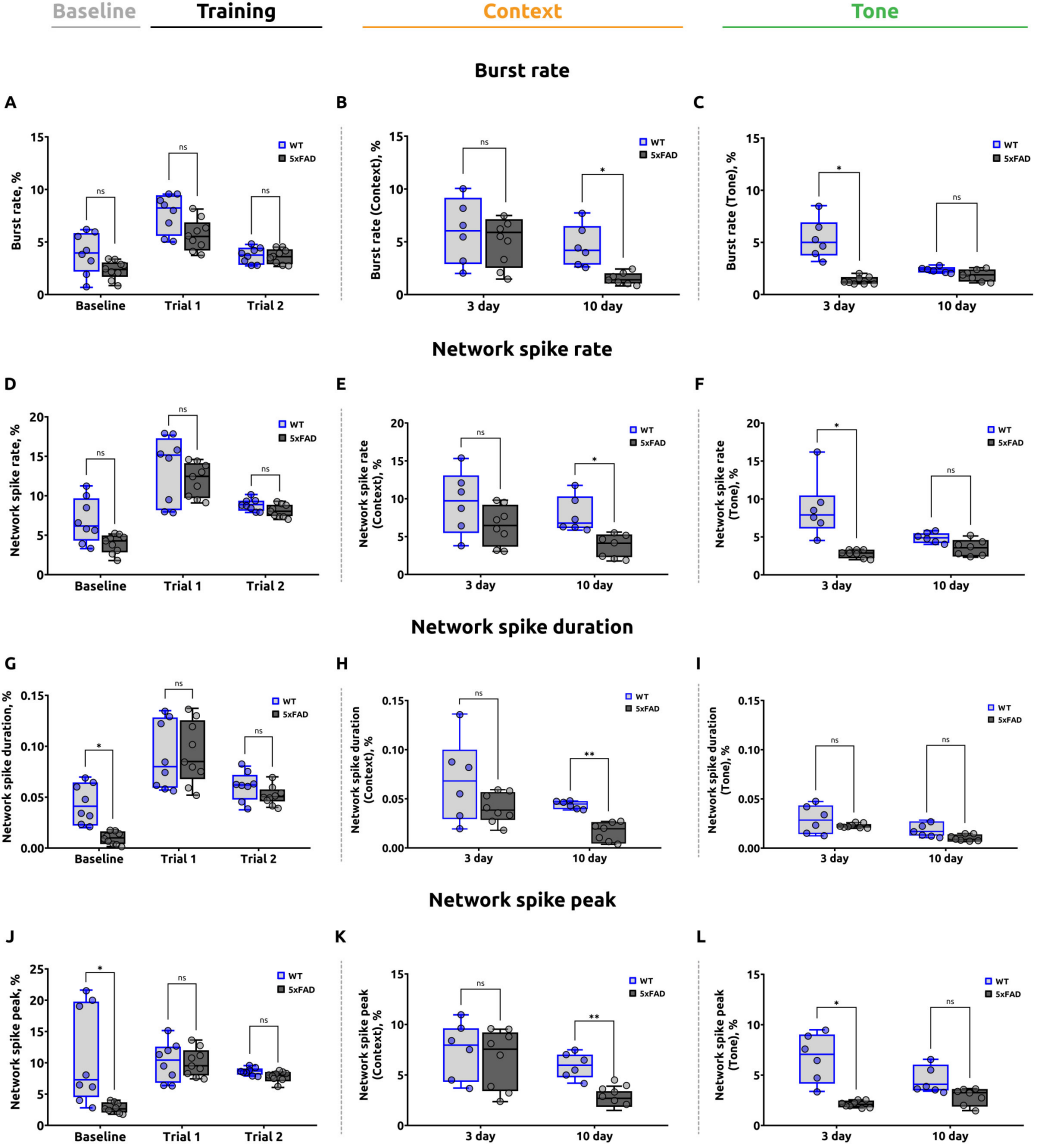
A summary of the data analysis conducted on the calcium imaging from the stratum pyramidale obtained from the fear conditioning test. **(A)** Burst rate during the baseline and training stages of the FCT (unpaired *t*-test, ns: *p* > 0.05). **(B)** Burst rate during the contextual test. On day 10, the 5xFAD mice exhibited a statistically significant reduction compared to WT mice (unpaired *t*-test for day 3 and 10, *p < 0.05, ns: *p* > 0.05). **(C)** Burst rate during the tone test was significantly reduced in 5xFAD mice on day 3 (Welch’s *t*-test for day 3, *p < 0.05; unpaired *t*-test for day 10, ns: *p* > 0.05). **(D)** Network spike rate (NSR) during the baseline and training stages of the experiment (unpaired *t*-test, ns: *p* > 0.05). **(E)** NSR during the contextual test. The data demonstrate a reduction in the 5xFAD mice on day 10, (unpaired *t*-test for day 3 and 10, *p < 0.05). **(F)** NSR during the tone test. The data demonstrated that the 5xFAD mice exhibited a reduced NSR on day 3 (Welch’s *t*-test for day 3, *p < 0.05; unpaired *t*-test for day 10, ns: *p* > 0.05). **(G)** Network spike duration (NSD) during the baseline and training stages of the experiment. A statistically significant reduction was observed in the 5xFAD mice during the baseline stage (Welch’s *t*-test for baseline, *p < 0.05; unpaired *t*-test for trial 1 and trial 2, ns: *p* > 0.05). No significant differences were identified during the training sessions. **(H)** NSD during the contextual test. The data indicated a reduction in the 5xFAD mice on day 10 (Welch’s *t*-test for day 3, ns: *p* > 0.05; unpaired *t*-test for day 10, **p < 0.01). **(I)** NSD during the tone test (Welch’s *t*-test for day 3, ns: *p* > 0.05; unpaired *t*-test for day 10, ns: *p* > 0.05). **(J)** Network spike peak (NSP) during the baseline and training stages of the experiment. A noteworthy decline was evident in the 5xFAD mice during the baseline phase, with no discernible discrepancy during the training sessions (Welch’s *t*-test for baseline, *p < 0.05; unpaired *t*-test for trial 1 and trial 2, ns: *p* > 0.05). **(K)** NSP during the contextual test. The data indicate that the NSP is lower in 5xFAD mice on day 10 (unpaired *t*-test for day 3 and day 10, **p < 0.01, ns: *p* > 0.05). **(L)** NSP during the tone test. The data demonstrate that the NSP is significantly lower in 5xFAD mice on day 3, with no significant difference on day 10 (Welch’s *t*-test for day 3, *p < 0.05; unpaired *t-test* for day 10, ns: *p* > 0.05). The data are presented as box-and-whisker plots: the line within the box represents the median, the box boundaries indicate the 25th and 75th percentiles, and whiskers show the minimum and maximum values (excluding outliers).

Subsequently, the data were analyzed in NeuroActivityToolkit in order to calculate a number of parameters, including burst rate, network spike rate, network spike duration, and network spike peak (see Section “2 Materials and methods”). The alterations in each of these parameters were analyzed during the evaluation of the baseline fear levels, the training sessions (trial 1 and trial 2), and on days 3 and 10. During the presentation of the CS, the analysis was performed in both the conditioning chamber (without the US and CS) and the altered conditioning chamber. The results are presented in Figure 4.

#### 3.2.1 Burst rate

The parameter “burst rate” was employed for the purpose of evaluating the number of calcium events per neuron across the entire network during specific stages of the FCT. On the second day of testing, during the assessment of baseline fear levels, no significant differences were identified between wild-type mice (3.865 ± 0.824) and 5xFAD mice (2.316 ± 0.360) (*p* = 0.1158) (Figure 4A).

In trial 1, the mean burst rate for the 5xFAD group was 5.618 ± 0.648, while the mean burst rate for the WT group was 7.755 ± 1.030. Nevertheless, no statistically significant differences were identified between the two groups (*p* = 0.0998). In trial 2, the mean burst rate was 3.599 ± 0.376 for the 5xFAD group and 3.664 ± 0.377 for the WT group. No statistically significant differences were identified between the two groups (*p* = 0.9078).

On the third day of the FCT, no statistically significant differences (*p* = 0.7002) were found in the burst rate parameter in 5xFAD mice (5.184 ± 1.296) compared to WT mice (6.033 ± 1.653) when assessing the fear response to a specific spatial context (Figure 4B). Nevertheless, on day 10, a significant reduction in the burst rate was observed in the 5xFAD group (1.507 ± 0.263) in comparison to the WT group (4.568 ± 0.912) (*p* = 0.0122).

A significant decrease in the burst rate parameter (*p* = 0.0110) was observed in 5xFAD mice (1.355 ± 0.155) compared to wild-type mice (5.264 ± 0.892) on day 3 of the FCT in the altered conditioning chamber (Figure 4C). However, on day 10, there was no statistically significant difference (*p* = 0.1013) in the burst rate parameter in 5xFAD mice (1.850 ± 0.234) compared to WT mice (2.370 ± 0.133).

#### 3.2.2 Network spike rate

The network spike rate (NSR) parameter was used to estimate the proportion of active neurons in the corresponding interval of the fear conditioning test. No significant differences (*p* = 0.0879) were found between 5xFAD (3.975 ± 0.588) and WT (6.827 ± 1.345) mice during the baseline fear assessment (Figure 4D). In trial 1, the mean NSR in the 5xFAD group was 12.027 ± 1.020, compared to 13.209 ± 1.560 in the WT group. In trial 2, the mean NSR was 8.105 ± 0.352 for the 5xFAD group and 8.847 ± 0.328 for the WT group. There were no significant differences between groups in either trial 1 (*p* = 0.5777) or trial 2 (*p* = 0.1540).

On the third day of the test, there were no statistically significant differences (*p* = 0.2817) in the network spike rate in 5xFAD mice (6.449 ± 1.423) compared to WT mice (9.371 ± 1.915) when assessing the fear response to a specific spatial context (Figure 4E) in a standard conditioning chamber. On day 10, however, a significant reduction (*p* = 0.0105) in the network spike rate was observed in 5xFAD mice (3.859 ± 0.695) in comparison to WT mice (7.872 ± 0.973).

In the conditioned stimulus tone test, a decreased (*p* = 0.0150) NSR was found in Alzheimer’s disease mice on day 3 (2.779 ± 0.257) compared to wild-type mice (8.620 ± 1.623) (Figure 4F). However, no significant differences (*p* = 0.0574) in network spike rate between 5xFAD (3.524 ± 0.510) and WT (4.846 ± 0.309) mice were detected at day 10.

#### 3.2.3 Network spike duration

Next, the network spike duration (NSD) parameter was estimated, which characterizes the period of time during which the number of simultaneously active neurons exceeds a given threshold (threshold = 0.8). During the baseline fear assessment, the NSD in 5xFAD mice was significantly (*p* = 0.0108) lower (0.010 ± 0.003) than in wild-type mice (0.043 ± 0.009). In the meantime, both groups’ mice showed no significant differences in the network spike duration parameter (*p* = 0.9782 for trial 1 and *p* = 0.3138 for trial 2). In trial 1, the value of network spike duration was 0.090 ± 0.012 in 5xFAD and 0.089 ± 0.012 in WT groups (Figure 4G). In trial 2, the NSD was 0.052 ± 0.004 for 5xFAD and 0.060 ± 0.007 for WT mice.

On day 3 of the contextual test, there were no significant differences (*p* = 0.1726) between the values of network spike duration in 5xFAD mice (0.040 ± 0.006) compared to WT mice (0.069 ± 0.017) (Figure 4H). On day 10 of the contextual test, network spike duration was significantly lower in 5xFAD (0.017 ± 0.004) compared to WT (0.044 ± 0.002) (*p* = 0.0014).

In the altered conditioning chamber, on days 3 (*p* = 0.3324) and 10 (*p* = 0.0584) of the conditioned tone test, there were no significant differences in network spike duration between the WT (0.029 ± 0.006 on day 3 and 0.019 ± 0.003 on day 10) and 5xFAD (0.023 ± 0.001 on day 3 and 0.010 ± 0.002 on day 10) groups (Figure 4I).

#### 3.2.4 Network spike peak

Subsequently, the parameter network spike peak (NSP), which represents the maximum number of neurons that are simultaneously active during a specified recording period within a given time interval, was evaluated.

During the baseline fear assessment, the NSP was significantly (*p* = 0.0445) lower in 5xFAD mice (2.825 ± 0.389) than in WT mice (10.053 ± 2.854) (Figure 4J). However, during training (trial 1 and trial 2), mice in both groups did not show significant differences in NSP (*p* = 0.7696 for trial 1 and *p* = 0.0591 for trial 2). In trial 1, the mean network spike peak value was 9.902 ± 1.053 in 5xFAD and 10.408 ± 1.195 in WT groups. In trial 2, the mean NSP was 7.768 ± 0.398 in 5xFAD and 8.683 ± 0.216 in WT.

On day 3 of the contextual test, when assessing the fear response to a specific spatial context, there was no statistically significant difference (*p* = 0.7540) in the network spike peak parameter in 5xFAD mice (6.559 ± 1.361) compared to WT mice (7.166 ± 1.285) (Figure 4K). The mean NSP was significantly lower in the 5xFAD group (2.633 ± 0.395) on day 10 of the contextual test in comparison to the WT group (5.909 ± 0.553) (*p* = 0.0013).

On day 3 of the conditioned stimulus tone test, NSP was significantly lower in AD mice (2.123 ± 0.169) compared to wild-type mice (6.748 ± 1.280) (*p* = 0.0352). However, when assessing the fear response to the tone in an altered chamber, there was no significant difference (*p* = 0.0517) in NSP in 5xFAD mice on day 10 of the tone test (2.876 ± 0.372) compared to WT mice (4.538 ± 0.707) (Figure 4L).

### 3.3 Recording of local field potentials in the hippocampal alveus

In addition to behavioral observations and calcium imaging, local field potentials in the hippocampal alveus (hippocampus white matter) were analyzed in the same animals that underwent the fear conditioning test with simultaneous calcium visualization. This analysis was facilitated by the use of a flexible microelectrode integrated with a gradient lens. Recordings were conducted in a two-channel mode with a sampling frequency of 1000 Hz.

**FIGURE 5:**
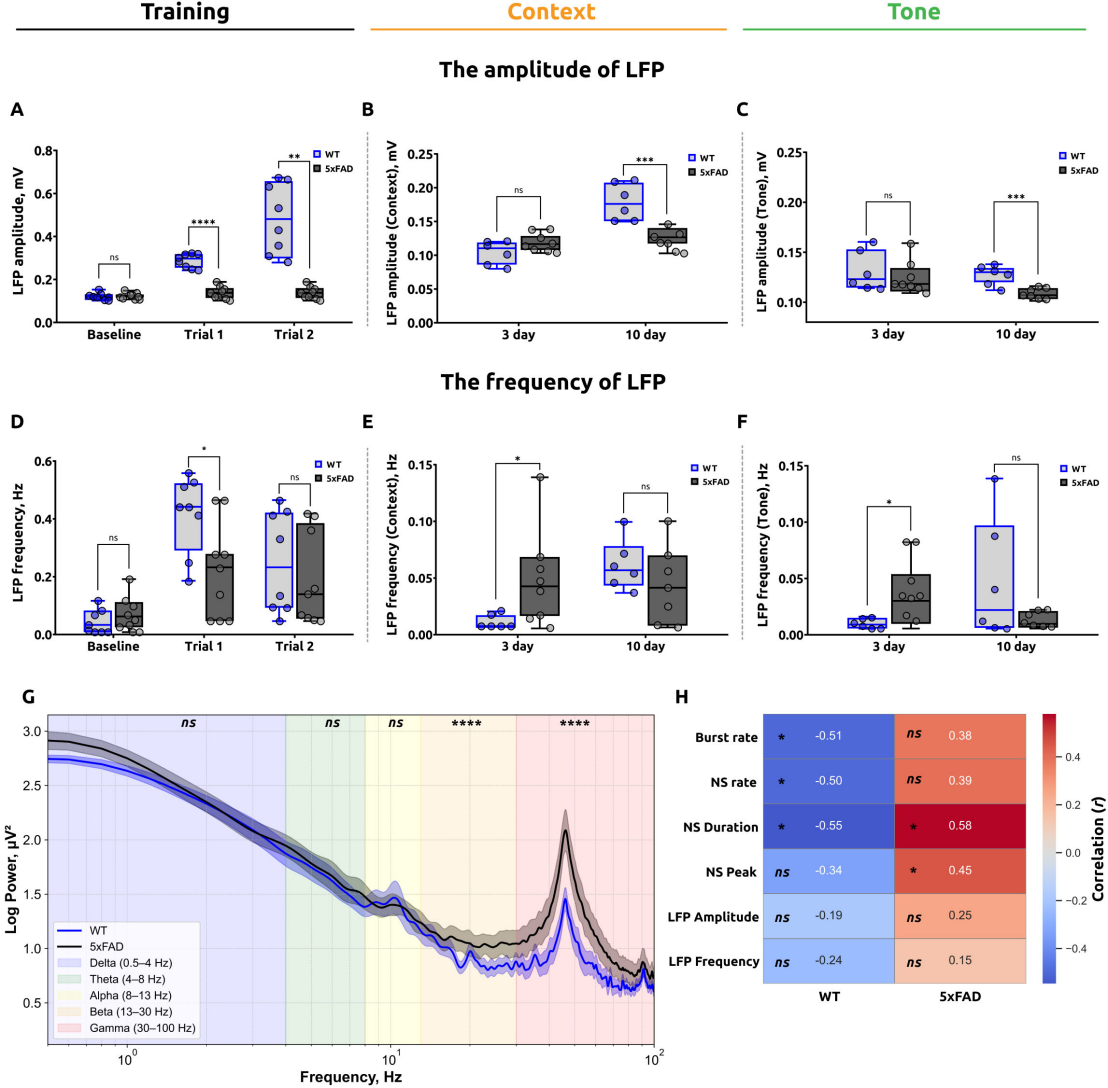
Summary of local field potential (LFP) analysis during the fear conditioning test (FCT) and correlation analysis. **(A)** LFP amplitude during the baseline and training stages of the FCT. The data demonstrate that there is no statistically significant difference between the WT and 5xFAD mice at the baseline level. However, during the training stage, a significant reduction in amplitude was observed in the 5xFAD mice (unpaired *t*-test for baseline and trial 1, ns: *p* > 0.05 and ****p < 0.0001, respectively; Mann-Whitney test for trial 2, **p < 0.005). **(B)** LFP amplitude in the contextual test. The data demonstrate that the 5xFAD mice exhibited a significant reduction on day 10 in comparison to the WT mice (unpaired *t*-test for day 3 and day 10, ns: *p* > 0.05, ***p < 0.001). **(C)** LFP amplitude in the tone test at day 10 is reduced in 5xFAD mice (unpaired *t*-test for day 3 and day 10, ns: *p* > 0.05, ***p < 0.001). **(D)** The frequency of LFPs recorded during the baseline and training stages of the experiment. The data suggest that WT mice exhibited a higher frequency of LFPs during trial 1 (unpaired *t*-test for baseline, trial 1 and trial 2, *p < 0.05, ns: *p* > 0.05. **(E)** The frequency of LFPs in the contextual test. On day 3, the frequency of LFPs was significantly higher in 5xFAD mice; however, no significant difference was observed on day 10 (Mann-Whitney test for day 3, *p < 0.05; Welch’s *t*-test for day 10, ns: *p* > 0.05). **(F)** The frequency of LFPs in the tone test. On day 3, the frequency of LFPs was significantly higher in 5xFAD mice; however, on day 10 no significant difference was observed (Welch’s *t*-test for day 3 and day 10, *p < 0.05, ns: *p* > 0.05). The data are presented as box-and-whisker plots: the line within the box represents the median, the box boundaries indicate the 25th and 75th percentiles, and whiskers show the minimum and maximum values (excluding outliers). **(G)** Power spectral density of raw LFP activity in the hippocampal alveus, calculated using 10-s time windows on the second day of the FCT for WT mice and 5xFAD mice. The data are presented as the average FFT of LFPs across all mice, with the shaded areas indicating the SEM. **(H)** Results of correlation analysis of the number of freezing episodes for WT (left) and 5xFAD (right) mice at each stage of the test with calcium imaging parameters (burst rate, network spike rate, network spike duration, network spike peak) and LFP characteristics (amplitude, frequency). The number in each cell is the corresponding Pearson’s correlation coefficient (*r*), the color reflects its sign and magnitude (scale on the right), *p < 0.05, ns: *p* > 0.05. In WT mice, a moderate negative correlation was found between the number of freezing episodes and calcium parameters: Burst rate, NSR, NSD. In 5xFAD mice, a moderate positive correlation was found between the number of freezing episodes and the parameters: NSD and NSP.

The spectral power analysis (Figure 5G) in the frequency range of 0.5–100 Hz revealed differences in neural activity between 5xFAD and WT mice. Five major frequency bands were analyzed: Delta (0.5–4 Hz), Theta (4–8 Hz), Alpha (8–13 Hz), Beta (13–30 Hz), and Gamma (30–100 Hz). In the Delta frequency band (0.5–4 Hz), the mean power in 5xFAD mice was 2.32 ± 0.08 μV^2^, which did not significantly differ from WT mice (2.27 ± 0.07 μV^2^; *p* = 0.5704). Similarly, in the Theta frequency band (4–8 Hz), no significant differences were observed, with mean power values of 1.68 ± 0.03 μV^2^ in 5xFAD and 1.61 ± 0.03 μV^2^ in WT mice (*p* = 0.6608). In the Alpha frequency band (8–13 Hz), the mean power was 1.36 ± 0.01 μV^2^ in 5xFAD mice compared to 1.35 ± 0.02 μV^2^ in WT mice, and this difference was also not statistically significant (*p* = 0.8737). Conversely, significant differences were identified in the Beta (13–30 Hz) and Gamma (30–100 Hz) frequency bands. In the Beta band, 5xFAD mice exhibited a mean power of 1.06 ± 0.01 μV^2^, significantly higher than that of WT mice (0.90 ± 0.01 μV^2^; *p* < 0.0001). Similarly, in the Gamma band, the mean power was 1.08 ± 0.02 μV^2^ in 5xFAD mice compared to 0.81 ± 0.01 μV^2^ in WT mice, with this difference also being statistically significant (*p* < 0.0001, Mann-Whitney test).

Furthermore, the electrophysiological data that was analyzed encompassed both the amplitude (mV) and the frequency of local field potentials. The results of this analysis are presented in Figure 5. The assessment of baseline fear levels revealed no statistically significant differences (*p* = 0.7600) in LFP amplitude between 5xFAD mice (0.124 ± 0.003) and WT mice (0.122 ± 0.007) (Figure 5A). In trial 1, the mean LFP amplitude for WT mice was 0.290 ± 0.017, which was significantly higher than that for 5xFAD mice, which was 0.138 ± 0.009 (*p* < 0.0001). In trial 2, the mean LFP amplitude for WT mice was 0.479 ± 0.096, which was also significantly higher than that for 5xFAD mice (0.134 ± 0.009; *p* = 0.0040).

On the third day of the test, when assessing the fear response to unconditioned room context in the standard conditioning chamber, no significant differences (*p* = 0.0929) in LFP amplitude were observed in 5xFAD mice (0.119 ± 0.003) compared to WT mice (0.105 ± 0.009) (Figure 5B). However, on day 10 of the test, a significant difference was observed in the mean LFP amplitude between the 5xFAD and WT mice. The mean LFP amplitude in the 5xFAD mice (0.126 ± 0.006) was found to be significantly lower (*p* = 0.0006) than that observed in the WT mice (0.178 ± 0.016).

The results observed in the altered conditioning chamber (Figure 5C) were comparable. On day 3 of the test, there was no significant difference (*p* = 0.6216) in LFP amplitude between mice with the Alzheimer’s disease model (0.124 ± 0.008) and wild-type (0.130 ± 0.011). On day 10 of the test, a significant reduction in LFP amplitude was observed in 5xFAD mice (0.108 ± 0.002) in comparison to WT mice (0.129 ± 0.004) (*p* = 0.0006).

Subsequently, the frequency of LFPs (Hz) was evaluated at key intervals throughout the fear conditioning test. At the baseline fear level assessment (Figure 5D), no significant difference was observed in the frequency of LFPs between 5xFAD mice (0.069 ± 0.013) and WT mice (0.046 ± 0.016; *p* = 0.3379). In trial 1, the mean frequency of LFPs in WT mice was 0.411 ± 0.056, which was significantly higher than in 5xFAD mice, which exhibited a mean of 0.211 ± 0.047 (*p* = 0.0185). In trial 2, the mean frequency of LFPs in WT mice was 0.279 ± 0.095, which was not significantly different from 5xFAD mice (0.186 ± 0.083; *p* = 0.6126).

On the third day of the test, the frequency of local field potentials in 5xFAD mice (0.048 ± 0.010) was found to be significantly higher (*p* = 0.0243) than in WT mice (0.011 ± 0.003) when evaluating the fear response to unconditioned room context in the standard conditioning chamber (Figure 5E). However, on day 10 of the contextual test, there was no significant difference (*p* = 0.2206) in the mean frequency of LFPs in 5xFAD mice (0.041 ± 0.010) compared to WT mice (0.060 ± 0.011).

Similar results were observed in the modified conditioning chamber (Figure 5F). On day 3 of the test with the conditioned stimulus (Tone), Alzheimer’s disease mice demonstrated significantly higher frequency of local field potentials (0.035 ± 0.009) compared to wild-type mice (0.010 ± 0.003) (*p* = 0.0193). However, on day 10 of the test with the conditioned stimulus (Tone), the frequency of LFPs in 5xFAD mice (0.012 ± 0.003) was not significantly different (*p* = 0.1857) from WT mice (0.046 ± 0.022).

### 3.4 Correlation analysis

The results of the correlation analysis (Figure 5H) in WT mice revealed a moderate negative correlation between the number of freezing episodes and the following calcium imaging parameters: the burst rate (*r* = −0.505, *p* = 0.0487), network spike rate (*r* = −0.502, *p* = 0.0493), and network spike duration (*r* = −0.546, *p* = 0.0412). In contrast, the correlation between freezing behavior and the network spike peak parameter was statistically insignificant (*r* = −0.337; *p* = 0.0779). There was also no significant correlation between behavioral and electrophysiological data: Amplitude of LFPs (*r* = −0.189; *p* = 0.2891), Frequency of LFPs (*r* = −0.239; *p* = 0.2396).

In 5xFAD mice, a moderate positive correlation was observed between the number of freezing episodes and the NSP parameter (*r* = 0.453, *p* = 0.0491) as well as the NSD parameter (*r* = 0.579, *p* = 0.0309). However, no statistically significant correlation was found for burst rate (*r* = 0.377; *p* = 0.1269) and network spike rate (*r* = 0.392; *p* = 0.1167). There was also no significant correlation with electrophysiologic data: Amplitude of LFPs (*r* = 0.250; *p* = 0.2290), Frequency of LFPs (*r* = 0.148; *p* = 0.3316).

## 4 Discussion

The present study investigated alterations in neural activity during fear conditioning in a 5xFAD mouse model of Alzheimer’s disease 5–6 months of age. This was achieved through the utilization of a combination of *in vivo* one-photon calcium imaging of CA1 hippocampal neurons and simultaneous electrophysiological recordings from the hippocampal alveus.

### 4.1 Fear conditioning test

An assessment of associative learning and memory in Alzheimer’s disease models (5xFAD) mice and wild-type (WT) mice, was conducted utilizing the Fear Conditioning Test (FCT). The findings revealed significant discrepancies in the behavioral patterns of the two mouse groups on both the third and tenth days of the FCT, indicating substantial deficits in associative learning and memory in the 5xFAD mice.

On the third day of FCT, the early cellular phase of memory consolidation was investigated. In the first hours after training, the stability of the formed memory is mainly ensured by post-translational mechanisms – receptor phosphorylation, kinase activation, and other rapid protein modifications (Johansen et al., 2011). However, 4–6 h after training, signaling cascades are activated, triggering the first wave of *de novo* protein synthesis: local translation of mRNA genes of immediate early response and synthesis of structural proteins necessary for maintaining long-term potentiation and increasing synaptic stability begin in dendrites (Raven et al., 2021). The tenth day of the test reflects the transition to the stage of systemic consolidation. By this point, a transcription-dependent wave of plasticity is realized, accompanied by the remodeling of dendritic spines and the beginning of functional reorganization of engrams (Dudai, 2004; Wiltgen and Silva, 2007). Protein synthesis-dependent and morphological changes already provide long-term memory stabilization, but complete fear conditioning memory consolidation can take up to 3–4 weeks (Wiltgen and Silva, 2007).

#### 4.1.1 Contextual text and the role of the hippocampus

On the third day of the contextual test, wild-type mice displayed a more pronounced fear response to the conditioned stimulus in comparison to 5xFAD mice. This may be attributed to the fact that the association between the neutral stimulus (CS) and the aversive stimulus (US) was still recent, which contributed to the pronounced fear response in WT mice. In contrast, 5xFAD mice demonstrated a reduction in freezing behavior at this stage, suggesting an impairment in contextual memory, which is highly dependent on the hippocampus (Frankland et al., 2004; Matus-Amat et al., 2004; Einarsson et al., 2015; Huiskamp et al., 2020). These findings are consistent with those of previous studies which demonstrate that hippocampal function is significantly affected in Alzheimer’s disease, particularly with regard to the formation and retrieval of contextual memory (Sun et al., 2019). Furthermore, it has been demonstrated that the explicit memory function, as evaluated by contextual fear conditioning, begins to decline in 5xFAD mice at approximately 5 months of age. This decline has been observed to coincide with synaptic dysfunctions of the hippocampus, including reduced basal synaptic transmission and deficits in long-term potentiation (Devi and Ohno, 2010).

On the tenth day of the contextual fear conditioning test, when long-term memory was assessed, the differences between the groups became even more pronounced. WT mice exhibited a robust fear response, indicating successful consolidation and long-term memory retention (Mizuno et al., 2012). In contrast, the level of freezing response (freezing) was significantly lower in mice of the 5xFAD line, which may indicate abnormalities in long-term plasticity processes, including changes in gene expression and structural changes in synapses (Mast et al., 2017). Furthermore, the reduced freezing response at day 10 in 5xFAD mice may indicate not only impairments in long-term memory formation but also a deficiency in its maintenance, resulting in the accelerated fading of conditioned fear (Kimura et al., 2010).

#### 4.1.2 Tonal test

On the third day of the tone test, the 5xFAD mice demonstrated a reduction in freezing behavior compared to the WT mice. This discrepancy may reflect broad disruptions in the neural circuitry underlying fear conditioning, which includes the amygdala, prefrontal cortex (PFC) and other modulatory networks regulating the amygdala-PFC circuit, such as the midbrain dopaminergic system in the ventral tegmental area and the locus coeruleus noradrenergic system, which are both degraded in AD (Vorobyov et al., 2019). Although the amygdala is indeed a critical area for forming and maintaining associations between auditory stimuli and aversive experiences (Wilensky et al., 1999), it functions in concert with these interconnected systems. In models of neurodegenerative diseases, such as 5xFAD mice, early pathological changes have been shown to occur in the amygdala, including neuronal loss and impaired synaptic transmission, leading to deficits in fear signaling and impaired formation of emotional memories (Schroeder and Shinnick-Gallagher, 2005). The aforementioned differences between WT and 5xFAD mice are further supported by data indicating that during the initial stages of learning, tone conditioning depends on rapid activation of the amygdala, where initial processing and memorization of conditioned fear occurs (Wemmie et al., 2003). In the absence of pathology, the amygdala interacts with the hippocampus and other brain structures to maintain long-term fear memories (Park et al., 2022).

The severity of freezing responses to the conditioned tone in WT mice may exceed the early contextual response, since associative LTP is induced in the basolateral amygdala (BLA) within a few hours after training, ensuring the consolidation of the tonal trace (Rogan et al., 1997). At the same time, in 5xFAD mice, Aβ accumulates in the BLA by 6 months of age, and BLA-CA1 projection abnormalities are observed (Xiong et al., 2024), which correlates with the fact that 5xFAD mice show a pronounced deficit in the cued fear conditioning test (Yang et al., 2017).

Additionally, a comparison was performed to evaluate the differences (Δ) in freezing behavior between WT and 5xFAD mice on days 3 and 10 during the tone test (Δ_3_^tone^ and Δ_10_^tone^) and the contextual test (Δ_3_^context^ and Δ_10_^context^). No statistically significant differences were observed between these values, indicating a pronounced reduction in freezing behavior in 5xFAD mice compared to WT mice during both the tone and contextual tests on days 3 and 10. These findings suggest a consistent deficit in associative learning and memory in 5xFAD mice.

### 4.2 Alterations to the calcium signaling of stratum pyramidale

One of the principal objectives of our research was to employ a combined methodology comprising *in vivo* calcium imaging and electrophysiological recording of local field potentials. This methodology enabled the simultaneous evaluation of alterations in hippocampal neuronal calcium activity and local field potentials in the hippocampal alveus during the animal’s behavioral activity. The calcium imaging results presented in the present study revealed significant alterations in the neuronal activity of hippocampal CA1 pyramidal cells in a mouse model of Alzheimer’s disease (5xFAD) in comparison to control animals (WT).

On the second day of FCT, WT mice demonstrated higher baseline calcium activity compared to 5xFAD mice, suggesting that the 5xFAD model is characterized by reduced basal neural or emotional reactivity (Padua et al., 2024). A decrease in calcium activity during baseline recording may indicate a reduction in the encoding of contextual features. Previously, it was shown that 5xFAD mice exhibit reduced calcium levels compared to WT in CA1 neurons during rest and unreliable spatial encoding as early as 4 months of age, leading to degradation of associations between objects and their locations (Zhang et al., 2023). During the first CS and in the first post-shock period, WT mice exhibited a marked increase in *ΔF/F* compared to 5xFAD mice, potentially reflecting impaired processing of the CS or reduced engagement with the task in AD model mice (Crouzin et al., 2013).

Interestingly, during the second presentations of the CS, 5xFAD mice displayed a level of activity comparable to that of wild-type mice and even superior in the second post-shock period. This observation may indicate the activation of compensatory mechanisms or task-specific recruitment of neural resources (Westi et al., 2024).

On the third day of the contextual test, 5xFAD mice exhibited higher calcium activity compared to WT mice, which may reflect aberrant neural mechanisms including compensatory overactivation (Palop and Mucke, 2016).

On the third day of the tone test, significantly reduced *ΔF/F* values during tone stimulation in 5xFAD mice support altered neural circuit responses under stimulus-driven conditions, consistent with findings of disrupted network oscillations and impaired information processing in AD models (Cassani et al., 2018).

On the tenth day, during contextual recall, 5xFAD mice had lower activity compared to WT mice, suggesting to progressive impairments in memory retention or retrieval. This finding is consistent with studies demonstrating progressive synapse loss and hippocampal dysfunction in late stages of AD pathology (Arendt, 2009; Braak and Del Tredici, 2015). In the tone test, WT mice sustained higher activity levels during the tone cue, underscoring the diminished capacity of 5xFAD mice to engage and sustain neural responses over time.

Furthermore, differences between WT and 5xFAD mice were observed in calcium activity parameters, including burst rate, network spike rate, network spike duration, and network spike peak. In particular, on the third and tenth days of the experiment, a reduction in parameters such as burst rate, network spike rate, and network spike peak was observed, which provided evidence of these alterations. These findings are consistent with those of previous studies which have reported decreased neuronal activity and disrupted spatial coding in the hippocampus of Alzheimer’s disease model mice (Zhang et al., 2023). Furthermore, the findings may be associated with a progressive decline in synapse functionality and impaired neuronal plasticity, which have also been previously described in the literature (Wang et al., 2020).

At the stage of determining the baseline fear level, significant differences were observed only for the parameters of network spike duration and network spike peak. One potential explanation for this is that these parameters were more sensitive to variations in the activity of neuronal ensembles between wild-type and 5xFAD mice. Specifically, NSD reflects changes in the duration of synchronous neuronal activity, while NSP is responsive to prominent bursts of coordinated network activity (Gerasimov et al., 2023). The observed reduction in the network spike duration parameter in 5xFAD mice suggests a decrease in the overall duration of neural activity, which may indicate alterations in arousal or a reduction in synchrony in firing patterns in this brain region (Gerasimov et al., 2023). Further evidence of impaired neuronal network synchronization is the reduction in the network spike peak parameter in AD mice, which reflects the maximum number of simultaneously active cells during the entire recording period.

Calcium imaging was performed on the pyramidal layer of cells within the CA1 region of the hippocampus. The dorsal CA1 region of the hippocampus is crucial for the execution of spatial memory tasks (Sekeres et al., 2010). It is noteworthy that significant alterations in the contextual test were only discernible on day 10 of the test, when long-term spatial memory was evaluated. This is likely due to the fact that changes at the synapse level, including the strengthening and stabilization of synaptic connections (Mast et al., 2017), are required for long-term memory formation. As the calcium imaging was conducted at the level of the entire neuronal network, the alterations became more apparent on day ten, when abnormalities in these processes, including decreased synaptic plasticity and functional connectivity, became evident.

It is also noteworthy that in the tone test, where the influence of the amygdala-prefrontal cortex circuit is predominant, significant differences are observed only on the third day in the parameters burst rate, network spike rate and network spike peak. However, no significant differences in the parameter network spike duration were observed. On the third day, it is reasonable to hypothesize that the hippocampus, in concert with its interconnected systems associated with the processing and integration of novel information, including emotionally salient data, into long-term memory, is engaged in heightened activity (von Allmen et al., 2013; Kragel et al., 2020). The discrepancies between wild-type mice and mice with Alzheimer’s disease may be attributed to anomalies in the hippocampus (Frankland et al., 2004; Matus-Amat et al., 2004; Einarsson et al., 2015; Huiskamp et al., 2020).

On the tenth day, during the transition to the systemic consolidation stage (Wiltgen and Silva, 2007), the activity of the amygdala and hippocampus in relation to this memory may decrease, since some of the information has already been processed and consolidated into long-term memory (Inman et al., 2018). Consequently, hippocampal calcium activity on the tenth day of the tone test may not show such pronounced differences because its function at this stage is primarily related to storage rather than active information processing (Fukushima et al., 2021). On day 10 during the presentation of the conditioned stimulus, no significant differences in calcium parameters were detected between wild-type and AD model mice, providing indirect corroboration of the hypothesis under discussion.

The reason for the absence of differences in network spike duration between WT and 5xFAD mice in the tone test remains unclear. The lack of notable discrepancies in network spike duration, which is indicative of the length of time during which neurons are synchronously active, may be attributed to the possibility that, despite an increase in overall activity, synchronization within the neuronal network may be impaired or unstable at the initial stages of the neurodegenerative process. Nevertheless, the absence of notable discrepancies in network spike duration on day ten, despite the occurrence of these processes, indicates that further investigation is required to comprehensively elucidate this phenomenon.

### 4.3 Alterations to the hippocampal LFP alveus

In our hands, the present study demonstrated significant alterations in neural oscillatory activity in 5xFAD mice compared to WT, characterized by increased power in both the Beta (13–30 Hz) and Gamma (30–100 Hz) frequency bands. This finding is in contrast to earlier studies reporting reduced Gamma oscillations in 5xFAD mice and other models of Alzheimer’s disease (Verret et al., 2012; Iaccarino et al., 2016; Zhen et al., 2017). Gamma oscillations, which are associated with synaptic integrity, inhibitory interneuron function, and cognitive processes, are typically reduced due to amyloid-beta-mediated synaptic toxicity and network dysfunction (Buzsaki and Wang, 2012).

However, a key difference between our study and others is the recording site: we measured neural activity from the hippocampal alveus. The alveus, which contains axons of pyramidal cells projecting to subcortical targets, may exhibit distinct oscillatory dynamics compared to other hippocampal regions, such as the CA1 or dentate gyrus, commonly studied in AD models. This anatomical specificity could explain the divergence in Gamma oscillation findings, as oscillatory patterns can vary significantly depending on the exact location of the recordings (Colgin, 2016).

Furthermore, while the Beta and Gamma bands exhibited significant alterations, no significant differences were found in the Delta, Theta, and Alpha frequency bands. Research indicates that lower-frequency bands often remain stable in the early stages of neurodegeneration, with alterations becoming more pronounced in advanced stages (Engels et al., 2016; Koelewijn et al., 2017; Maturana-Candelas et al., 2020). Additionally, a multi-compartment model of β-amyloid-induced Ca^2 +^ imbalance predicts an increase in phase dispersion at an early stage, while a distinct decrease in theta oscillations occurs later (Kaushik et al., 2022). Classic studies of the generation of theta oscillations emphasize the important role of Ca^2 +^ signals in CA1 pyramidal neurons (Buzsaki, 2002; Vertes, 2005), but the fact that electrophysiological recording was performed in the hippocampal axonal output layer rather than directly in the CA1 pyramidal layer also explains the absence of pronounced changes in theta rhythm in 5xFAD mice.

During the baseline fear stage, no significant differences in the amplitude and frequency of LFP were observed between wild-type mice and an Alzheimer’s disease model, despite the differences detected by calcium imaging. This discrepancy may be attributed to differences in the sensitivity of the employed calcium imaging and LFP recording methods. Calcium imaging provides insight into intracellular processes associated with the activation and function of individual neurons, whereas LFP recordings offer a comprehensive representation of electrical activity on a larger scale (Chemin et al., 2002).

It has been demonstrated that alterations in axon activity may not necessarily correlate with changes in calcium activity in pyramidal neuron bodies. This is attributed to the distinctive characteristics of the hippocampal alveus, which is primarily composed of axons rather than neuron bodies (Ofer et al., 2024). This distinction is significant because alterations in axon function may not directly correlate with changes in pyramidal neuron bodies, which are particularly susceptible in the initial stages of neurodegeneration (Wines-Samuelson et al., 2010).

The hippocampal alveus plays a crucial role in the transmission of information from the hippocampus to other regions of the limbic system and the cerebral cortex. The axons that constitute the hippocampal alveus are directed toward the vault (fornix), which then transmits signals to a number of different brain regions, including the hypothalamus and the amygdala (Daitz and Powell, 1954).

The absence or low activity of processes related to memory, learning, and spatial orientation during the baseline fear detection phase may explain the lack of significant differences in LFP amplitude and frequency between WT and 5xFAD mice. This suggests that in the baseline phase, critical information transfer processes via the hippocampal alveus to other brain regions may not be fully engaged (Xiong et al., 2017).

It is notable that 5xFAD mice have been observed to exhibit axonopathy in various fibrous structures, including the hippocampal alveus. The severity of this pathology increases with age (Zhang et al., 2020). During training sessions, these 5xFAD mice exhibited a significant decrease in LFP amplitude and frequency compared to wild-type mice. This finding is consistent with the evidence of axonal degeneration observed in these mice.

It is noteworthy that, despite these changes in LFP, no significant differences were observed in the calcium imaging parameters at the same stages. This suggests that while axonal degeneration affects LFP, it may not directly correlate with alterations in calcium dynamics at these specific stages of learning and memory processing.

The absence of differences in LFP amplitude on day three in contextual and tone tests may be attributed to compensatory mechanisms of short-term synaptic plasticity, whereby neuronal ensembles endeavor to maintain functionality by increasing the frequency of LFPs. However, a further reduction in LFP amplitude on day ten indicates a depletion of these compensatory resources, which is accompanied by an impairment of cognitive function and memory (Durand et al., 2006; Zhang et al., 2020). A reduction in the amplitude of LFP in 5xFAD mice at day ten in both contextual and tone tests indicate the degradation of neural pathways (axonopathy) and a decline in cognitive function as the disease progresses (Mok et al., 2017; Trillaud-Doppia and Boehm, 2018; Zhang et al., 2020).

Finally, the results of the study indicate that impaired neuronal activity and synchronization in the hippocampus and hippocampal alveus in the Alzheimer’s disease mouse model are associated with the onset of cognitive deficits related to learning and memory. These findings highlight the necessity for further research aimed at elucidating the mechanisms of neurodegeneration and identifying potential therapeutic targets to address these impairments.

### 4.4 Correlation analysis

The results of the correlation analysis revealed fundamental differences in the direction of correlations between behavioral responses and measures of neural activity in WT and AD mice. In WT mice, a moderate negative correlation was observed between the number of freezing events and several calcium imaging parameters, including burst rate, network spike rate, and network spike duration. These findings suggest a tendency for neural activity to decrease as the behavioral response associated with fear or anxiety intensifies, potentially reflecting a more coordinated neural network (McEwen et al., 2016). Conversely, no relationship with the NSP parameter, which characterizes the maximum number of simultaneously active neurons, was found, which may be due to the balanced operation of the neural network (Cohen and Maunsell, 2010).

In contrast, 5xFAD mice demonstrated a moderate positive correlation between the number of freezing events and the calcium imaging parameters NSP and NSD. This finding suggests that as the behavioral response intensified, the maximum number of active neurons increased, and the duration of their activity was prolonged. These changes may reflect an uncoordinated regulation of these processes in the AD model mice (Verret et al., 2012; Busche and Konnerth, 2016). Furthermore, the relationship with burst rate, which characterizes the number of calcium events per neuron, and network spike rate, which is used to estimate the proportion of active neurons throughout the network, was insignificant, which tells us that despite the increase in the dynamics of active neurons, the overall network activity changed little, which also indicates the uncoordinated operation of neuronal ensembles (Busche et al., 2008; Styr and Slutsky, 2018).

The correlation between behavior and electrophysiological data was statistically insignificant in both WT and 5xFAD mice. The amplitude and frequency of LFPs, which reflect the total activity of large populations of neurons and synapses, are likely sensitive to the overall brain state (healthy or pathological) but may not respond selectively to specific behavioral changes (Busche et al., 2008). Nonetheless, this study identified phenotypic differences in electrophysiological recordings between WT and AD model mice, underscoring distinct neural network dynamics associated with the disease state.

### 4.5 Limitation of the study

In light of the substantial findings, it is imperative to consider the limitations of the present study when interpreting the data and extrapolating it to more comprehensive contexts.

Firstly, although the 5xFAD transgenic mouse model of Alzheimer’s disease is widely recognized and utilized in neurodegenerative disease research, it is not without limitations. The model effectively replicates certain aspects of AD pathology, including plaque deposition and cognitive decline, within a relatively short period of time. However, the timeline and pattern of disease progression may not align perfectly with the natural course of AD in humans (Sadleir et al., 2018; Bundy et al., 2019). This discrepancy in disease progression dynamics between the 5xFAD model and human AD highlights the necessity for a cautious interpretation of research findings derived from this model, as the results may not fully reflect the processes occurring in the human disease (Milanovic et al., 2018).

The study employed both male and female mice as experimental subjects. However, recent studies have indicated that female 5xFAD mice exhibited a lower learning performance compared to males, suggesting a gender disparity in cognitive function in the 5xFAD mouse model of Alzheimer’s disease (O’Leary and Brown, 2022). Moreover, gender-based differences in olfactory recognition have been observed (Bouter et al., 2021), along with a more pronounced hippocampal pathology in 5xFAD females (Bundy et al., 2019). The presented data highlight the necessity of considering gender differences in cognitive studies for more accurate data analysis.

In addition, sensory processing deficits, particularly in visual function, may impact behavior in the 5xFAD model. Studies have reported retinal dysfunction in these mice, which could interfere with visual cue recognition in context-based tasks, such as fear conditioning (Criscuolo et al., 2018). This limitation suggests a potential confounding effect of sensory impairment on observed freezing behavior, potentially influencing memory assessments. Future studies could address this by implementing preliminary vision screening and adjusting behavioral protocols to control for sensory deficits. Such measures would help isolate the specific cognitive impacts of neurodegeneration from sensory-related artifacts.

Moreover, the implementation of single-photon fluorescence microscopy and electrophysiological techniques is constrained by the intrinsic limitations of the methodologies employed. Although single-photon fluorescence microscopy permits the examination of neuronal calcium activity *in vivo*, it exhibits a restricted spatial resolution in comparison to two-photon microscopy. This may result in a reduction in data accuracy (Liu et al., 2020; Li et al., 2022). Additionally, single-photon calcium imaging data can be affected by local background fluctuations, which can impact the observed neuronal fluorescence (Yoshida et al., 2018). Furthermore, electrophysiological recordings, particularly those employing miniaturized wireless systems, may demonstrate constraints in sensitivity and resolution, which can affect the precision of detecting the activity of individual neurons and neuronal groups (Buzsaki et al., 2012).

Another significant limitation is the potential impact of chronic microelectrode and miniscope implantation on animal behavior and neuronal activity. Despite the implementation of measures to minimize stress in the animals during the experiment, it is not feasible to entirely negate the possibility of the implant influencing the outcomes of the experiment (Yoshida et al., 2018).

It is important to note that the results of this study were obtained from a small sample of mice, which limits the statistical power of the analysis and requires caution when interpreting the data and extending the findings to the general population. Further studies utilizing larger samples and diverse models and techniques are essential to substantiate the findings and facilitate their broader applicability (Button et al., 2013).

Another limitation of our study is the use of the adeno-associated viral vector AAV5.Syn.GCaMP6f.WPRE.SV40, which induces GCaMP expression in both excitatory and inhibitory neurons (Nathanson et al., 2009). This complicates precise determination of each cell type’s contribution to the observed changes in calcium activity. Furthermore, potential interactions between different neuron types may lead to complex effects that are difficult to account for with the current experimental design. Possible solutions to this issue include the use of neuron-specific promoters or transgenic models to achieve selective expression of calcium indicators in either excitatory or inhibitory neurons (Sohn et al., 2017; Ravindra Kumar et al., 2020; Radhiyanti et al., 2021). Also an important limitation to the simultaneous analysis of alveus electrophysiological recording data and calcium imaging of the CA1 region of the hippocampus is that alveus fibers contain axons originating from the DG and from CA3, CA2, CA1, and subiculum pyramidal neurons (Knowles and Schwartzkroin, 1981), so these signals may only indirectly reflect CA1 cell activity. In addition, there was no pharmacological confirmation of the exact cellular origin of the signals recorded.

It is also worth noting that although our study found a significant increase in electrophysiological activity in the gamma and beta frequency ranges in 5xFAD mice, we did not record LFPs in naive mice.

In view of the aforementioned limitations, it is essential to exercise caution when interpreting the findings of this study and to pursue further research in order to gain a deeper understanding of the neurodegenerative processes associated with Alzheimer’s disease.

## 5 Conclusion

We used *in vivo* calcium imaging with wireless electrophysiology to study neuronal activity and synaptic dynamics in the hippocampus of 5xFAD mice. The results show that associative learning and memory are severely impaired in 5xFAD mice, as evidenced by changes in calcium signaling and disruption of local field potentials in CA1 and alveolus.

These results demonstrate that advanced imaging and recording can detect early neurophysiological changes in Alzheimer’s disease. The decline in neuronal activity and spatial coding in the 5xFAD model means that we need to detect and intervene early in AD. Addressing the limitations of this study will allow us to better understand the mechanisms of cognitive loss in neurodegenerative diseases. This can then be used to design specific therapies to preserve cognition.

## Data Availability

The original contributions presented in this study are included in this article/supplementary material, further inquiries can be directed to the corresponding author.

## Footnotes

1
https://github.com/bothlab/pomidaq

2
https://github.com/syntalos/syntalos

3
https://github.com/AutoHotkey/AutoHotkey

4
https://www.anaconda.com/download

5
https://github.com/denisecailab/minian

6
https://mamba.readthedocs.io/en/latest/installation/mamba-installation.html

7
https://github.com/spbstu-applied-math/NeuroActivityToolkit
